# Functional and regulatory conservation of the soybean ER stress-induced DCD/NRP-mediated cell death signaling in plants

**DOI:** 10.1186/s12870-016-0843-z

**Published:** 2016-07-12

**Authors:** Pedro A. B. Reis, Paola A. Carpinetti, Paula P.J. Freitas, Eulálio G.D. Santos, Luiz F. Camargos, Igor H.T. Oliveira, José Cleydson F. Silva, Humberto H. Carvalho, Maximiller Dal-Bianco, Juliana R.L. Soares-Ramos, Elizabeth P. B. Fontes

**Affiliations:** Departamento de Bioquímica e Biologia Molecular, Universidade Federal de Viçosa, Viçosa, MG Brazil; National Institute of Science and Technology in Plant-Pest Interactions, Bioagro, Universidade Federal de Viçosa, Viçosa, MG Brazil

**Keywords:** Programmed cell death, Abiotic stresses, ER stress, N-rich proteins, NAC transcription factors, Vacuolar processing enzyme, VPE, NRPs, BiP, Binding protein

## Abstract

**Background:**

The developmental and cell death domain (DCD)-containing asparagine-rich proteins (NRPs) were first identified in soybean (*Glycine max*) as transducers of a cell death signal derived from prolonged endoplasmic reticulum (ER) stress, osmotic stress, drought or developmentally-programmed leaf senescence via the GmNAC81/GmNAC30/GmVPE signaling module. In spite of the relevance of the DCD/NRP-mediated signaling as a versatile adaptive response to multiple stresses, mechanistic knowledge of the pathway is lacking and the extent to which this pathway may operate in the plant kingdom has not been investigated.

**Results:**

Here, we demonstrated that the DCD/NRP-mediated signaling also propagates a stress-induced cell death signal in other plant species with features of a programmed cell death (PCD) response. *In silico* analysis revealed that several plant genomes harbor conserved sequences of the pathway components, which share functional analogy with their soybean counterparts. We showed that GmNRPs, *GmNAC81*and *VPE* orthologs from Arabidopsis, designated as *AtNRP*-*1*, *AtNRP*-*2*, *ANAC036* and *gVPE*, respectively, induced cell death when transiently expressed *in N. benthamiana* leaves. In addition, loss of *AtNRP1* and *AtNRP2* function attenuated ER stress-induced cell death in Arabidopsis, which was in marked contrast with the enhanced cell death phenotype displayed by overexpressing lines as compared to Col-0. Furthermore, atnrp-1 knockout mutants displayed enhanced sensitivity to PEG-induced osmotic stress, a phenotype that could be complemented with ectopic expression of either *GmNRP*-*A* or *GmNRP*-*B*. In addition, AtNRPs, *ANAC036* and *gVPE* were induced by osmotic and ER stress to an extent that was modulated by the ER-resident molecular chaperone binding protein (BiP) similarly as in soybean. Finally, as putative downstream components of the NRP-mediated cell death signaling, the stress induction of *AtNRP2*, *ANAC036* and *gVPE* was dependent on the *AtNRP1* function. BiP overexpression also conferred tolerance to water stress in Arabidopsis, most likely due to modulation of the drought-induced NRP-mediated cell death response.

**Conclusion:**

Our results indicated that the NRP-mediated cell death signaling operates in the plant kingdom with conserved regulatory mechanisms and hence may be target for engineering stress tolerance and adaptation in crops.

**Electronic supplementary material:**

The online version of this article (doi:10.1186/s12870-016-0843-z) contains supplementary material, which is available to authorized users.

## Background

Environmental changes and extreme conditions, such as temperature variations, drought and salinity, adversely affect plant growth and cause major yield loss of agriculturally relevant crops worldwide. However, plants do not passively accept these abiotic stresses and, therefore, have developed sophisticated mechanisms for perception, transduction and adaptive responses to cope with the environmental stressors and to restore the cellular homeostasis under stress conditions [1, 2]. In eukaryotic cells, the stress signaling systems allow intensive and integrate communications not only between the cell surface and the extracellular environment but also among intracellular organelles, which can accommodate adaptive responses. The understanding of the plant stress signaling systems along with the distinctions between detrimental effects and adaptive advantage is crucial for engineering superior crops.

The endoplasmic reticulum (ER) is a key signaling organelle involved in the activation of cellular stress responses in eukaryotic cells. One such well-characterized signaling event is the unfolded protein response (UPR), which is activated to cope with the disruption of ER homeostasis that results in the accumulation of unfolded or misfolded proteins in the lumen of the organelle [3, 4]. In mammalian cells, UPR is transduced as a tripartite module through the ER membrane receptors (i) protein kinase-like ER kinase (PERK), (ii) inositol-requiring transmembrane kinase and endonuclease 1α (IRE1) and (iii) activating transcription factor 6 (ATF6) [3]. Upon disruption of ER homeostasis, plant cells activate UPR through IRE1 homologs (IRE1a and IRE1b, in Arabidopsis) and membrane–tethered bZIP transcription factors (bZIP28 and bZIP17 in Arabidopsis), which are functionally related to the mammalian ATF6 [4, 5]. The mRNA of a third class of ER membrane-associated UPR transducer, bZIP60, serves as a substrate for the endonuclease activity of IRE1a/IRE1b [6]. IRE1a/b activation by ER stress mediates an unconventional splicing of the unspliced bZIP60 mRNA (bZIP60u) to generate an alternatively spliced transcript (bZIP60s), which lacks the transmembrane-encoded sequences and hence is translated into a soluble protein to activate UPR inducible promoters in the nucleus. ER stress also triggers the release of bZIP17/bZIP28 from the ER membrane [7]. Upon stress, bZIP17 and bZIP28 move from the ER membrane to the Golgi where they are proteolytically cleaved by SP1 and SP2 allowing the bZIP domain to be translocated to the nucleus [5–7]. Recently, a plasma membrane-associated member of the plant-specific NAC domain-containing TF family, AtNAC062, has also been described as a relevant player in regulating UPR downstream gene expression [8]. Therefore, in plants, the UPR operates via IRE1a/IRE1b-bZIP60, SP1/SP2-bZIP17/bZIP28 and AtNAC062 modules to coordinately up-regulate ER-resident molecular chaperones and activate the ER-associated degradation protein system [4–6, 9]. However, if ER stress is sustained and UPR fails to restore ER homeostasis, a cell death signal is activated. Persistent ER stress has been shown to trigger both ER–stress specific and shared PCD (programmed cell death) signaling pathways elicited by other death stimuli [10–12].

A plant-specific ER stress-induced cell death response is mediated by the ER membrane-tethered NAC089 transcription factor [13]. In response to ER stress, NAC089 is relocated to the nucleus to control the expression of downstream genes involved in PCD, such as *NAC094*, *METACASPASE 5* (*MC5*) and *BCL*-*2 ASSOCIATED ATHANOGENE* (*BAG6*). In addition to the NAC089-mediated cell death response, the Arabidopsis G protein β-subunit1 [AGB1] was firstly reported as a positive regulator of ER stress-induced cell death [14], but contrasting results were later reported by Chen and Brandizzi [15]. More recently, AGB1 was shown to function as a cell death positive regulator as mutations in *AGB1* suppressed the cell death response in bir1-1 and in transgenic plants overexpressing *SUPPRESSOR OF BIR1* (*SOBIR1*) [16].

A distinct plant-specific, ER stress-shared cell death response is the ER and osmotic stress-integrated signaling, which converges on developmental cell death domain (DCD)-containing N-rich proteins (NRPs) to transduce a cell death signal with hallmarks of PCD [17, 18]. The expression of *DCD*/*NRP* is controlled by the ER and osmotic stress-induced transcription factor (TF) GmERD15, which specifically binds to the DCD/NRP promoters to activate the transcription of these genes [19]. Induction of *DCD*/*NRP* activates a signaling cascade that culminates with the induction of plant-specific TFs GmNAC81 and GmNAC30 [20, 21], which form heterodimers to fully transactivate the vacuolar processing enzyme (VPE) promoter [21]. VPE exhibits caspase-1-like activity and induces plant-specific PCD, mediated by collapse of the vacuole [21, 22]. Therefore, DCD/NRP, GmNAC081, GmNAC030 and VPE are involved in a plant-specific regulatory cascade that integrates osmotic stress–and ER stress–induced PCD. Because DCD/NRP was the first component to be discovered, this stress–induced transduction pathway is often referred to as the NRP–mediated cell death signaling [23].

As a branch of the ER stress response that connects with other environmentally induced responses, the NRP–mediated cell death signaling pathway may allow for the versatile adaptation of cells to different stresses [11]. Accordingly, we have previously showed that this pathway is activated by drought and the modulation of this signaling event by the constitutive expression of the ER–resident molecular chaperone binding protein (BiP) promotes a better adaptation of transgenic lines to drought [24, 25]. *BiP* overexpression also increased tolerance of soybean transgenic seedlings to tunicamycin, an inducer of ER stress, and to PEG, which induces osmotic stress [18]. In soybean, BiP attenuates the propagation of the stress–induced cell death signal by modulating the expression and activity of the components of the cell death pathway *GmNRP*–*A*, G*mNRP*–*B*, *GmNAC81* and *VPE* [18, 26].

In spite of the relevance of the DCD/NRP–mediated signaling as a versatile adaptive response to multiple stresses, mechanistic knowledge of the pathway is lacking and the extent to which this pathway may operate in the plant kingdom has not been investigated. Here, we showed first that the DCD/NRP–mediated cell death components are represented in both dicotyledonous and monocotyledonous genome and, like in soybean, they function to propagate a cell death signal in response to ER and osmotic stress in Arabidopsis. Using reverse genetic, the characterized elements were sequentially ordered in the signaling pathway. Furthermore, we showed that Arabidopsis BiP attenuates the DCD/NRP–mediated cell death signaling and thereby confers tolerance to drought in Arabidopsis, suggesting that conserved regulatory mechanisms are responsible for the BiP–mediated increases in water stress tolerance in plants.

## Results

### The components of the DCD/NRP–mediated cell death signaling are widely distributed in the plant kingdom

The previously characterized soybean genes of the NRP–mediated cell death signaling were used as prototypes for the identification of homologs in the genomes of *Arabidopsis thaliana*, *Carica papaya*, *Citrus sinensis*, *Cucumis sativis*, *Glycine max*, *Manihot esculenta*, *Phaseolus vulgaris*, *Solanum lycopersicum*, *Solanum tuberosum*, *Triticum aestivum*, *Oryza sativa* and *Zea mays*. For each signaling module component, we selected the five most related components of each plant species to construct phylogenetic trees using Bayesian inference.

**Fig. 1:**
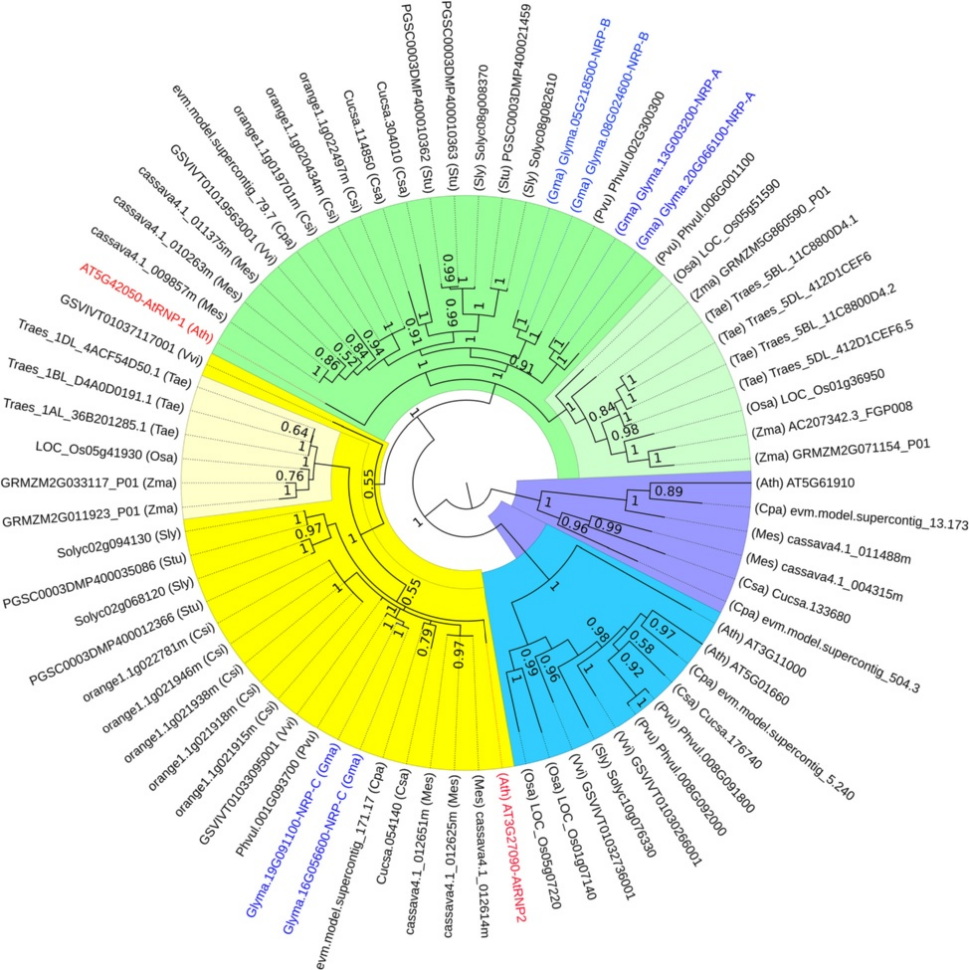
Phylogenetic analysis of GmNRP–like genes. The amino acid sequences of NRP-like proteins were recovered from TAIR (http://arabidopsis.org/) and Phytozome v10.3 databases and aligned using MUSCLE. Phylogenetic trees were constructed using Bayesian inference performed with MrBayes v3.2.2 with mixed amino acid substitution model (Blosum). The analyses were carried out running 20.000.000 generations and excluding the first 5.000.000 generations as burn–in. GmNRPs from soybean is depicted in blue and from Arabidopsis in red. Background colors demark clusters and sub–clusters formed by NRP homologs. The three–letter abbreviation in parenthesis preceding the nomenclature of the NRP homologs denotes the plant species, such as Ath: *Arabidopsis thaliana*, Cpa: *Carica papaya*, Csi: *Citrus sinensis*, Csa: *Cucumis sativis*, Gma: *Glycine max*, Mes: *Manihot esculenta*, Pvu: *Phaseolus vulgaris*, Sly: *Solanum lycopersicum*, Stu: *Solanum tuberosum*, Tae: *Triticum aestivum*, Osa: *Oryza sativa* and Zma: *Zea mays*

A striking feature of the soybean genome is the retention of extended blocks of duplicated genes [27]. The six soybean *GmNRP* paralogs (in blue) were clustered in pairs, consistent with duplication events. *GmNRP*–*A* and *GmNRP*–*B* were more closely related to each other as they clustered together (green cluster) and differed largely from the *GmNRP*–*C* sequences (yellow cluster; Fig. 1). Both *GmNRP–A* and *GmNRP–B* are involved in the NRP–mediated cell death signaling and they displayed representative homologs in all plant species [17] (Fig. 1). Among the Arabidopsis *NRP* homologs, *AtNRP*–*1* (in red) and *AtNRP*-*2* (in red) displayed the highest sequence similarity to GmNRPs; *AtNRP1* (AT5G42050) clustered with *GmNRP*-*A* and *GmNRP*-*B*, whereas *AtNRP2* (AT3G27090) was close related to *GmNRP*-*C* from soybean. The Arabidopsis AtNRP1 has been described previously [28]. Like AtNRP1, AtNRP2 contains N-rich and DCD domains and belongs to the group I of DCD domain–containing proteins [29].

Although DCD/NRP–A and DCD/NRP–B have redundant and relevant functions in cell death signaling, it remains to be determined whether GmNRP–C also functions in the transduction pathway. The other three selected Arabidopsis NRP–like sequences formed two separate groups (blue and purple clusters), which may not represent *NRP* orthologs of soybean *GmNRP*–*A* and *GmNRP*–*B* due to the low similarity of sequence among them. In both *GmNRP*–*A*/*GmNRP*–*B*–based cluster (green) and *GmNRP*–*C* cluster (yellow), the *NRP* homologs formed sub–clades of monocotyledonous and dicotyledonous genes. The conservation of sequences of these NRP–like genes in other plant species is strongly suggestive of their functional importance and identities.

The execution of the cell death program has been proposed to occur through NRP–mediated induction of the *GmNAC81*–*GmNAC30*–*VPE* module [21]. Consistent with a duplication event, *GmNAC81* is clustered in pair with the paralog *GmNAC77* (see green cluster); whereas *GmNAC30* is represented by a small family in the soybean genome (Additional files 1 and 2) [6]. *GmNAC81* and *GmNAC77* form a unique clade (green) that encompasses at least one possible ortholog from each plant species, including monocotyledonous and dicotyledonous representatives and a single–copy gene from Arabidopsis (*ANAC036*/AT2G17040). The *GmNAC30*–based clade (Additional file 2, green) contains the five members of the soybean *GmNAC30* family (*GmNAC18*, *GmNAC22*, *GmNAC30*, *GmNAC35*, *GmNAC39*) and four homologs from Arabidopsis, in addition to representatives of all plant species. The other selected sequences that cluster separately from the *GmNAC81* and *GmNAC30* clades were not considered putative orthologs due to the low sequence identity and lack of functional characterization.

The *VPE* family has five representatives in the soybean genome [21]. Phylogenetic analysis revealed that four soybean *VPE* paralogs (in blue), and two Arabidopsis paralogs, *alphaVPE* and *gammaVPE*, formed a unique clade (green) that was separated from the fifth soybean *VPE*, Glyma01g05135, which clustered with monocotyledonous homologs (Additional file 3). In addition to high similarity of sequence, the Arabidopsis *alphaVPE* and *gammaVPE* display similar expression pattern and the encoded proteins exhibit caspase 1–like activity [30]. The four most closely related soybean VPEs display similar expression profiles during development and in response to stress and one of them, Glyma.14G092800, has been shown to be induced by GmNAC81 and GmNAC30 [21, 26]. The expression profiles and functions of more distantly related VPEs have not been examined. The high conservation of the components of the ER stress NRP–mediated cell death signaling among soybean and other dicotyledonous and monocotyledonous plant species suggests that this cell death signaling may be a general ER stress response in plants rather a specific transduction pathway in soybean.

We next examined whether the structural homology of the pathway components would reflect functional conservation of the cell death response in plants. The molecular tools for the characterization of this pathway in other plant species are still limited. In contrast, in the Arabidopsis model system, reverse genetic studies are possible to assign function and hierarchical order to components of signal transduction pathways. Therefore, we examined whether the stress–induced DCD/NRP–mediated signaling would function in Arabidopsis, integrating multiple stress signals into a cell death response, as described in soybean.

### Functional conservation of the stress–induced DCD/NRP–mediated cell death response in Arabidopsis

**Fig. 2:**
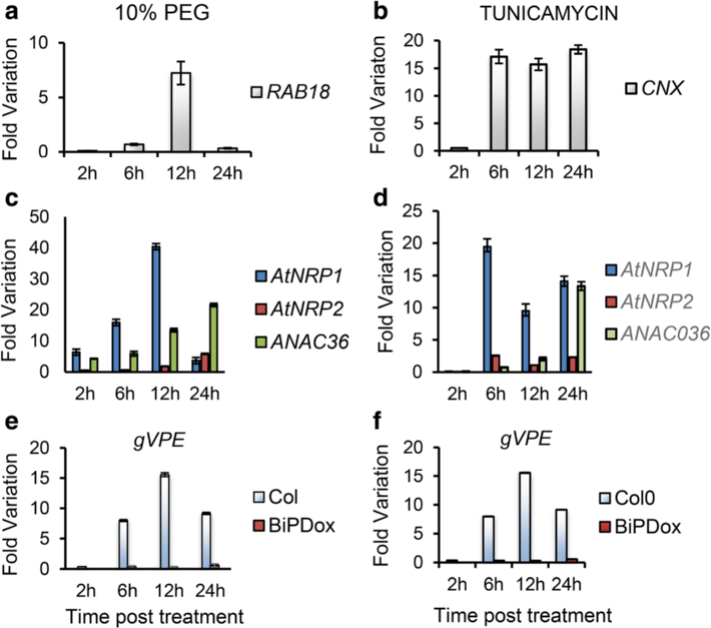
*AtNRP1*, *AtNRP2*, *ANAC036* and *gVPE* are induced by osmotic and ER stresses. Total RNA was isolated from 15 days–old Arabidopsis seedlings that had been treated with PEG (10 % w/v) or Tunicamycin (2,5 μg/mL) for 2 h, 6 h, 12 h and 24 h. H_2_O was used as control for PEG and DMSO for Tunicamycin. The transcript levels of selected genes, as indicated, were quantified by qRT–PCR. Gene expression was calculated using the 2^-ΔΔCt^ method and *UBQ5* as endogenous control. cDNAs were obtained from five biological replicates and validated individually. *RAB18* and *CNX* are osmotic stress and ER stress gene markers, respectively. (S.E., *n* = 5 biological replicates). Col denotes Col–0 (wild–type) line and BiPDox is Arabdidopsis transgenic lines ectopically expressing the soyBiPD gene. **a** PEG induction of *RAB18*. **b** Tunicamycin induction of calnexin (*CNX*). **c** PEG induction of *AtNRP1*, *AtNRP2* and *ANAC036*. **d** Tunicamycin induction of *AtNRP1*, *AtNRP2* and *ANAC036.* **e** PEG induction of *VPE*. **f** Tunicamycin induction of *VPE*

Soybean NRPs and *GmNAC81* are induced by the osmotic stress inducer PEG, and the inducer of ER stress, tunicamycin [17, 20]. Among a series of other stress inducers, the gene *AtNRP1* has also been shown to be induced by osmotic stress [28] and in response to the ER stress inducer tunicamycin [31, 32]. As putative components of the stress–induced DCD/NRP–mediated signaling that integrates a cell death signal in response to ER stress and osmotic stress, we examined whether *AtNRP2* and *ANAC036* would respond to these stresses. Fifteen days–old Arabidopsis seedlings (columbia background) were treated with PEG (10 % w/v) and tunicamycin (2,5 μg/mL) during 24 h and the gene expression was analyzed by qRT–PCR. The effectiveness of the stress treatments was monitored by analyzing the expression of the osmotic–stress marker *RAB18* gene and the ER stress marker *calnexin* (*CNX*) gene (Fig. 2a, b). Under these conditions, *AtNRP1*, *AtNRP2* and *ANAC036* were induced by osmotic stress (Fig. 2c) and ER stress (Fig. 2d), although with differences in their induction kinetics. *AtNRP1* displayed higher level of induction at 12 h after PEG treatment and at 6 h after tunicamycin treatment. *AtNRP2* was also induced by both treatments, although to a lower extent as compared to the expression of *AtNRP1* and exhibited a late kinetic of induction in response to PEG. *ANAC036* was induced with different kinetic from AtNRPs, reaching maximum induction at 24 h after PEG and tunicamycin treatment. We also monitored the tunicamycin and PEG induction of an Arabidopsis *VPE* ortholog [*gamma* (*g*) *VPE*], which has been shown to be the downstream component of the pathway that acts as the executioner of the cell death program (Fig. 1e, f) [21, 22, 30]. Like the other components of the pathway, *gVPE* was induced by osmotic stress (PEG) and ER stress (tunicamycin).

**Fig. 3:**
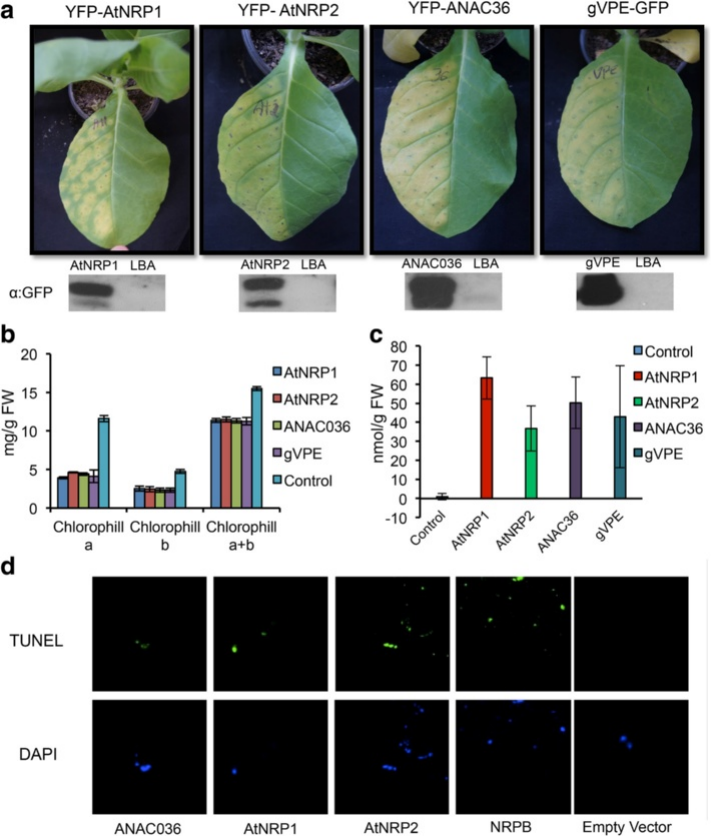
Arabidopsis AtNRP1, AtNRP2, ANA036 and VPE cause cell death *in planta*. **a** Phenotypes of agroinfiltrated leaves with the indicated genes. The left half of leaves from 3 weeks–old *N. benthamiana* were infiltrated with agrobacterium cells transformed with p35S: AtNRP1, p35S: AtNRP2, p35S: ANAC036 and p35S: VPE expression vectors. Pictures were taken 6 days after infiltration. The lower panel shows the immunoblottings of the agroinfiltrated proteins probed with anti–GFP serum. **b** Chlorophyll loss induced by *AtNRP1*, *AtNRP2*, *ANAC036* and *VPE* expression. Total chlorophyll, chlorophyll a and chlorophyll b were determined from the leaf sectors agroinfiltrated with the described DNA constructions. Values are given as mean ± S.E. from three biological replicates. **c** Lipid peroxidation induced by *AtNRP1*, *AtNRP2*, *ANAC036* and *VPE* expression. Leaf lipid peroxidation was monitored by determining the level of thiobarbituric acid–reactive compounds and expressed as the malondialdehyde content. error bars indicate the 95 % confidence interval based on a t–test (*p* < 0,05, *n* = 3). **d** Transient expression of DCD/NRP–mediated cell death signaling orthologs from Arabidopsis induces DNA fragmentation. Tobacco protoplasts were electroporated with the constructions carrying *AtNRP1*, *AtNRP2*, *ANAC036*, *NRP*–*B*, under control of 35S promoter or the empty vector, as a negative control. After 36 h of agroinfiltration, protoplasts from leaf sectors were submitted to TUNEL labeling. The nuclei were stained with DAPI

As putative components of the ER stress–and osmotic stress–integrating signaling pathway, we examined whether transient expression of *AtNRP1*, *AtNRP2*, *ANAC036* and *VPE* would activate a cell death program in tobacco leaves. After 7 days of agroinfiltration, the leaf sectors expressing *AtNRP1*, *AtNRP2*, *ANAC036* and *VPE* displayed a chlorotic phenotype with necrotic lesions as a result of massive cell death, as opposing to the green phenotype of the right half of the leaves, which was infiltrated with Agrobacterium alone (Fig. 3a, Additional file 4a, b, c). The transient expression of the transgenes (GFP–or YFP–fused proteins) was monitored by immunoblotting total protein from agroinfiltrated sectors with anti–GFP serum (Fig. 3a, lower panel) and by determining transcript accumulation via qRT–PCR (Additional file 5a, b, c, d). The expression of the positive control genes, *GmNRP*–*A* and *GmNRP*–*B*, also induced a chlorotic phenotype (Additional file 4d, e), contrasting with the remaining green phenotype displayed by the expression of a *BiP* gene, used for cell death inhibition (Additional file 4f). These phenotypes correlated with the chlorophyll loss in the agroinfiltrated sectors (Fig. 3b, Additional file 4g) and the extent of lipid peroxidation (Fig. 3c) and suggest a role for AtNRP1, AtNRP2, ANAC036 and VPE as effectors of a cell death response. This interpretation was further confirmed by applying the terminal deoxynucleotidyl transferase–mediated dUTP nick end labeling (TUNEL) assay for the *in situ* detection of DNA fragmentation in the *AtNRP1*–, *AtNRP2*–and *ANAC036*–expressing leaf sectors (Fig. 3d). The extensive cleavage of nuclear DNA is one feature of cell death. The nuclei of the leaf sectors that were transformed with the empty vector fluoresced intensely with DAPI and exhibited only TUNEL–negative nuclei. In contrast, the *AtNRP1*–, *AtNRP2*–and *ANAC036*–expressing samples had TUNEL–positive nuclei that displayed the same degree of staining as the *GmNRP*–*B*–expressing leaf sectors (Fig. 3d). These results suggest that *AtNRP1*, *AtNRP2* and *ANAC036* promote cell death when they are transiently expressed in tobacco leaves, a functional role reminiscent of the soybean components of the osmotic stress–and ER stress–induced cell death signaling pathway [18]. VPE has also been show to mediate PCD in plants [22]. VPE–dependent PCD pathway has been shown to operate in the immune response, in the responses to a variety of stress inducers, in leaf senescence and in the development of various tissues [21, 22, 30, 33].

**Fig. 4:**
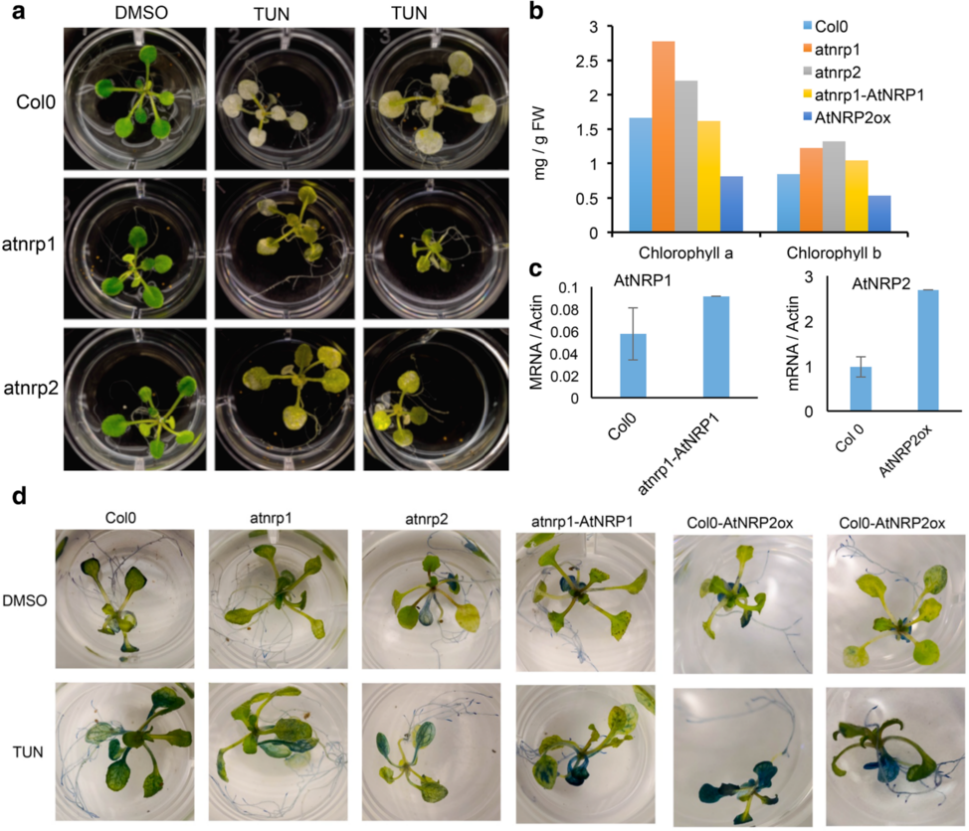
AtNRP1 and AtNRP2 are involved in ER stress–induced cell death in Arabidopsis. **a** Loss of *AtNRP1* and *AtNRP2* function attenuated ER–stress induced chlorophyll loss in Arabidopsis. ER stress was induced by transferring atnrp1 and atnrp2 seedlings to MS medium containing 5 μg/μLtunicamycin. Photography was taken 2 days after ER stress induction. **b** Chlorophyll content of knockouts, atnrp1–complementing and *AtNRP2*–overexpressing lines under ER stress. The chlorophyll content of seedlings from the genotypes, as indicated in the figure, was determined 24–h after tunicamycin treatment. **c** Expression levels of *AtNRP1* in complementing lines and *AtNRP2* in overexpressing lines. Total RNA was isolated from 7 days-old Arabidopsis seedlings, genotypes atnrp1 transformed with 35S: AtNRP1 and Col–0 transformed with 35S: AtNRP2. The transcript levels of *AtNRP1* or *AtNRP2*, as indicated, were quantified by qRT–PCR. Gene expression was calculated using the 2^-ΔCt^ method and *Actin* as endogenous control. Values are mean ± S.D. from three replicates. **d** Evans blue staining of Arabidopsis seedlings treated with 5 μg/mL tunicamycin or DMSO. Col–0, atnrp1, atnrp2, atnrp1–complementing line and *AtNRP2*–overexpressing lines were treated with tunicamycin for 24 h and stained with Evans blue

We next used reverse genetics to examine whether AtNRP1 and AtNRP2 were involved in an ER stress–induced cell death program in Arabidopsis. RT–PCR on RNA from atnrp1 or atnrp2 leaves detected no accumulation of the AtNRP1or AtNRP2 transcripts in the homozygous T–DNA insertion mutant, confirming it is atnrp1 or atnerp2 null alleles (Additional file 5e and f). The ER stress inducer tunicamycin has been shown to promote cell death in soybean and Arabdopsis leaves with hallmarks of senescence and PCD. Seedlings of atnrp1 and atnrp2 knockout lines were treated with the ER stress inducer tunicamycin and we monitored leaf yellowing and chlorophyll loss (Fig. 4b). After four days of treatment, the leaves of Col–0 were completely pale, whereas the leaves of atnrp1 and atnrp2 displayed green sectors, characteristic of chlorophyll integrity. This phenotype was associated with higher chlorophyll content in atnrp1 and atnrp2 stressed seedlings as compared with wild type stressed seedlings (Fig. 4c). Expression of *AtNRP1* in the atnrp1 mutant restored the wild type content of chlorophyll (see atnrp1 + *AtNRP1*) and overexpression of *AtNRP2* increased ER stress–induced chlorophyll loss, a phenotype consistent with enhanced cell death in overexpressing lines. Although we could select for *AtNRP1*–complementing lines in the atnrp1 background, we did not obtain *AtNRP1*–overexpressing lines; thereby, the overexpression studies were restricted to *AtNRP2*. These results were complemented with Evans blue staining of Arabidopsis seedlings under ER stress conditions, as a measurement of cell death (Fig. 4d). Loss of *AtNRP1* or *AtNRP2* function in atnrp1 and atnrp2 lines attenuated ER stress–induced cell death as compared to Col–0, which was in marked contrast with the enhanced cell death phenotype displayed by ER–stressed *AtNRP2*–overexpressing lines. Collectively, these results indicated that, similarly to the orthologs *GmNRP*–*A* and *GmNRP*–*B* from soybean, *AtNRP1* and *AtNRP2* are involved in ER stress–induced cell death in Arabidopsis.

**Fig. 5:**
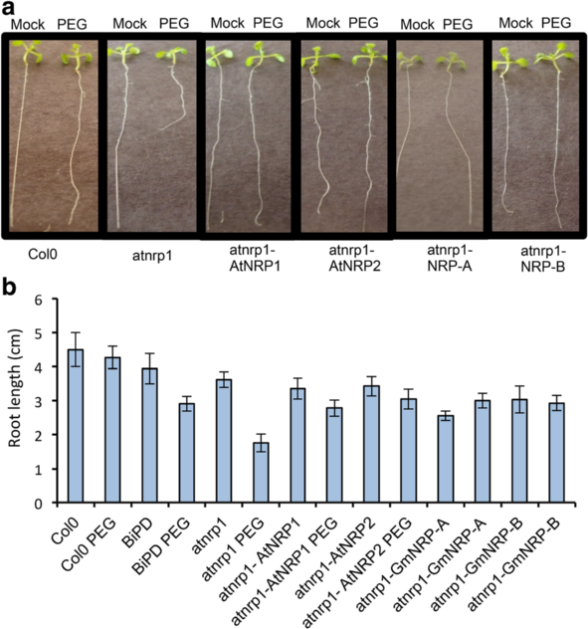
AtNRP2, NRP–A and NRP–B complement the enhanced sensitivity phenotype of root growth to osmotic stress displayed by the atnrp1 knockout line. **a** Complementation assays of the atnrp1 function. The knockout line atnrp1 was transformed with p35S: AtNRP1, p35S: AtNRP2, p35S: NRP–A and p35S: NRP–B and germinated in LS–agar plates with and without PEG (0,5 %) during 6 days. Seeds of Col–0 and atnrp1 lines were also germinated in LS–agar plates with and without PEG (0,5 %) and root length was measured at 6 days post–germination Photography was taken 6 days after germination under osmotic stress. **b** Measurement of root length from Col–0, atnrp1 null alleles and atnrp1–complementing lines. Error bars indicate the 95 % confidence interval based on a t–test (*p* < 0,05, *n* = 15)

To examine further the functional relatedness between soybean and Arabidopsis NRPs, we took advantage of the stress hypersensitive phenotype of atnrp1 null alleles (Salk_041306) for complementation assays. Inactivation of *AtNRP1* gene has been shown to cause a higher inhibition of seedling root growth under osmotic stress as compared to wild type seedlings [28]. Likewise, we found that PEG inhibited root growth to a higher extent in atnrp1 knockout seedlings than in wild–type seedlings (Fig. 5a). This phenotype was linked to the inactivation of the *AtNRP1* gene because expression of *AtNRP1* in the atnrp1 restored the wild type phenotype (Fig. 5a, b). In order to determine whether GmNRPs would replace the *AtNRP1* function, we transformed the knockout line with *GmNRP*–*A*, *GmNRP*–*B* and the Arabidopsis homolog *AtNRP2*, under the control of 35S promoter. Ectopic expression of *AtNRP2*, *GmNRP*–*A* and *GmNRP*–*B* reversed the *atnrp1* phenotype upon osmotic stress as the complemented transgenic lines displayed wilt type root growth under PEG (Fig. 5a, b). Collectively, these results further indicated that Arabidopsis and soybean NRPs are functionally related.

### The downstream components *AtNAC036* and *VPE* require the *AtNRP1* function for tunicamycin and PEG induction

**Fig. 6:**
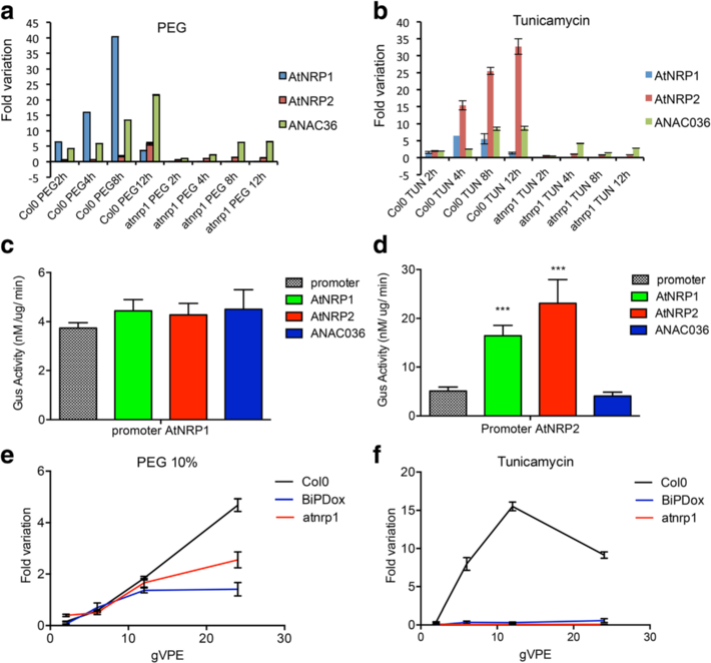
AtNRP1 is upstream of AtNRP2 and ANA036 in the stress–induced cell death response. **a** and **b** *AtNRP1*, *AtNRP2* and *ANAC036* expression in Col–0 and atnrp1 knockout line. Total RNA was isolated from 15 days–old Arabidopsis seedlings treated with **a** PEG (10 %) and **b** Tunicamycin (2,5 μg/mL) for 2 h, 6 h, 12 h and 24 h. H_2_O was used as control for PEG and DMSO for Tunicamycin. The transcript levels of selected genes were quantified by qRT–PCR. Gene expression was calculated using the 2^-∆∆Ct^ method using *UBQ5* as endogenous control. cDNAs were obtained from five biological replicates and validated individually. **c** and **d** Ectopic expression of *AtNRP1* activated the AtNRP2 promoter. Tobacco leaves were co–infiltrated with Agrobacterium carrying AtNRP1pro:βGUS **c** or AtNRP2pro:βGUS **d** in combination with YFP–AtNRP1, *AtNRP2* or YFP–ANAC036. Values represent β–Glucuronidase activity of three biological replicates and asterisks indicate statistical differences by the test t (*p* < 0,05). **e** and **f** *VPE* expression in Col–0, *BiP*–overexpressing lines and atnrp1 lines. Total RNA was isolated from 15 days–old Arabidopsis seedlings treated with **e** PEG (10 %) and **f** Tunicamycin (2,5 μg/mL) for 2 h, 6 h, 12 h and 24 h and the transcript level was monitored by qRT–PCR as described in a and b

GmNAC81 has been placed downstream of NRPs in the stress–induced NRP–mediated cell death signaling based on expression analysis and kinetics of the cell death activities of the pathway components [20]. Ectopic expression of *GmNRP*–*A* or *GmNRP*–*B* has been shown to activate the GmNAC81 promoter and to induce *GmNAC81* expression. Furthermore, stress induction of *GmNRP*–*B* and *GmNRP*–*A* genes precedes the induction of *GmNAC81* and GmNAC81–mediated cell death in tobacco leaves occurs with early kinetics, as expected from a downstream effector of the pathway. This sequential order of the components in the transduction signal pathway was confirmed in the Arabidopsis system by a reverse genetic approach and promoter transactivation assay (Fig. 6). Both *atnrp1* and Col–0 lines were treated with PEG and tunicamycin for 12 h and the extent of *AtNRP2* and *ANAC036* induction was determined by qRT–PCR (Fig. 6a, b). The expression levels of *AtNRP2* and *ANAC036* induced by tunicamycin or PEG were remarkably lower in *atnrp1* line compared to Col–0. Furthermore, *in the atnrp1* line, the stress induction of *ANAC036* gene was delayed. These results indicate that the full induction of *AtNRP2* and *ANAC036* by osmotic or ER stress requires the *AtNRP1* function. To confirm that AtNRP2 acts downstream of AtNRP1, we performed a GUS transactivation assay in tobacco leaves using the 2–kb 5′ flanking sequences of *AtNRP1* and *AtNRP2* genes fused to the GUS reporter. Transient expression of *AtNRP1*, *AtNRP2* or *ANAC036* did not activate the AtNRP1 promoter (Fig. 6c), whereas expression of *AtNRP*1 and *AtNRP2*, but not *ANAC036*, activated the AtNRP2 promoter (Fig. 6d). Collectively, these results placed AtNRP1 upstream of AtNRP2 and confirmed that ANAC36 is downstream of AtNRPs in the pathway. gVPE was also genetically linked to the stress–induced NRP–mediated cell death signaling because induction of *gVPE* by ER stress and osmotic stress required the *AtNRP1* function (Fig. 6e, f). This result confirmed the biochemical data that identified VPE as a downstream component in the NRP–mediated cell death response in soybean [21].

### *BiP* overexpression attenuates the expression of DCD/NRP–mediated cell death genes and promotes water stress tolerance in Arabidopsis

The stress–induced NRP–mediated cell death response has been shown to be modulated by BiP [17, 18]. Overexpression of *soyBiPD* (Glyma.05G219400.1.p) in soybean attenuates and delays the cell death response induced by osmotic stress, ER stress and drought, a phenotype that has been linked to the BiP–mediated increases in the water stress tolerance [18, 24]. Among the Arabidopsis BiP paralogs, AtBiP1 is most related to AtBiP2 (98 % amino acid sequence identity) and they share the highest sequence conservation with soyBiPD (91 % sequence identity), whereas AtBiP3 is 77 % identical to soyBiPD. Thereby, *AtBiP1* and *AtBiP2* genes were selected to examine whether the NRP–mediated cell death response in Arabidopsis would share similar regulatory mechanisms as in soybean. Then, we transformed Arabidopsis Col–0 with *soyBiPD* and also with the Arabidopsis BiP genes, *AtBiP1* and *AtBiP2*, and monitored the BiP attenuation of the stress–induced expression of pathway components. The ectopically expressed soybean BiPD protein accumulated to high levels in the independently transformed Arabidopsis T07, T10, T13, T23 lines and was correctly localized in microsomal fraction (Additional file 6a and b). UGPase was used as a cytosolic fraction–associated marker to demonstrate that soyBiPD was confined to the microsomal fraction (Additional file 6c). Likewise, Arabidopsis transformed with AtBiP1–GFP–HDEL and AtBiP2–GFP–HDEL fusions accumulated higher levels of BiP mRNA (Additional file 7a) and protein (Additional file 7b) than Col–0. Accumulation of BiP–GFP–HDEL was detected by immunoblotting total protein with anti–GFP serum (Additional file 7b, lower blot) and the endogenous BiP levels + fusion proteins were monitored with an anti–soyBiPD serum (upper blot). Like the endogenous BiPs (Additional file 7c, Col0), BiP–GFP–HDEL was correctly localized in the microsomal fraction (AtBiP1).

**Fig. 7:**
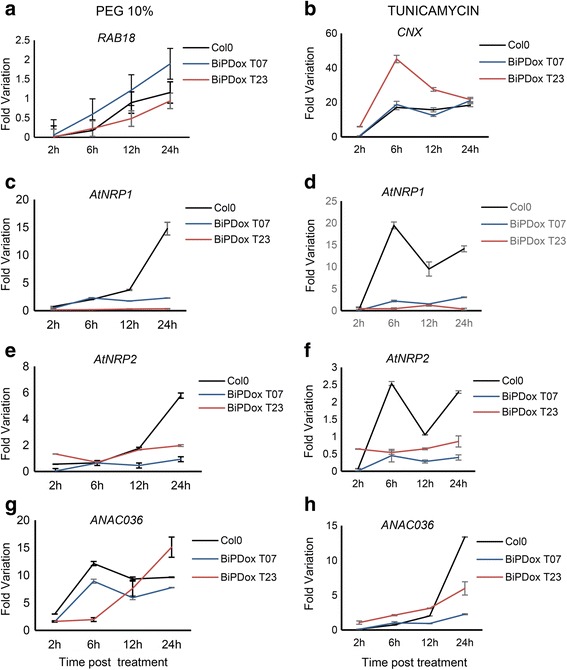
Overexpression of *soyBiPD* in Arabidopsis attenuates the PEG and tuncamycin–mediated induction of DCD/NRP–mediated cell death signaling genes. **a** and **b** *AtNRP1*, *AtNRP2* and *ANAC036* expression in Col–0 and *BiPD*–overexpressing lines. Total RNA was isolated from 15 days–old Arabidopsis plants treated with PEG (10 % w/v) and Tunicamycin (2,5 μg/mL) for 2 h, 6 h, 12 h and 24 h. H2O was used as control for PEG and DMSO for Tunicamycin. The transcript levels of selected genes were quantified by qRT–PCR. Gene expression was calculated using the 2^-ΔΔCt^ method and *UBQ5* as endogenous control. cDNAs were obtained from four biological replicates and validated individually. (S.E., *n* = 4 biological replicates). BiPDox T07 and BiPDox T23 are transgenic Arabidopsis lines independently transformed with the *soyBiPD* gene. **a**, **c**, **e**, **g** Time course of PEG-induced *RAB18*, *AtNRP1*, *AtNRP2* and *ANC036* in Col-0 and BiP-overexpressing lines. **b**, **d**, **f**, **h** Time course of tunicamycin-induced *CNX*, *AtNRP1*, *AtNRP2* and *ANC036* in Col-0 and BiP overexpressing lines

The induction of *AtNRP1*, *AtNRP2* and *ANAC036* by tunicamycin was lower in all *BiP*–overexpressing lines than in Col–0 (Fig. 7b and Additional file 7d). Likewise, PEG treatment induced the expression of *AtNRP1*, *AtNRP2* and *ANAC036* to a lower extent in BiPDox T07, BiPDox T23 lines and BiP1–overexpressing line than in Col–0 (Fig. 7a and Additional file 7e). These results confirmed that BiP also modulates the NRP–mediated cell death response in Arabidopsis.

**Fig. 8:**
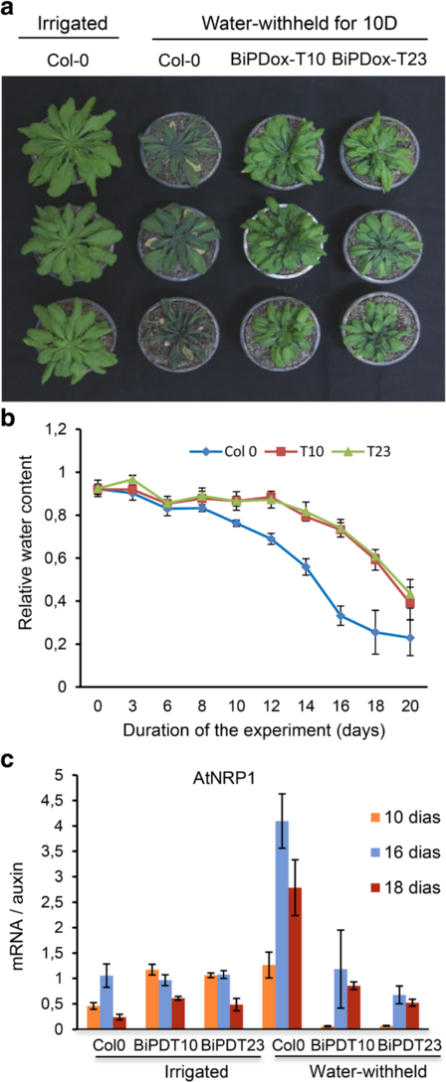
*BiPD*–overexpressing lines are more tolerant to water stress. **a** Transgenic lines under a water deficit regime. Arabidopsis plants, genotypes Col–0, BiPDox line T10 and BiPDox line T23 were grown in soil and water stress was induced by withholding irrigation for 20 days. Photography was taken 10 days after withholding irrigation. **b** Relative water content of wild–type and transgenic leaves exposed to the drought regime. **c** Expression of *AtNRP1* and *AtNRP2* during the water stress treatment. Total RNA was isolated from leaves of Col–0 and BiPDox lines at the indicated time points during the water stress treatment. The transcript levels of *AtNRP1* were quantified by qRT–PCR. Gene expression was calculated using the 2^-ΔΔCt^ method and *Actin* as endogenous control. cDNAs were obtained from five biological replicates and validated individually. Error bars indicate the 95 % confidence interval based on a t-test (*p* < 0,05)

The BiP–mediated attenuation of the stress–induced NRP–mediated cell death response has been linked to its capacity to confer tolerance to drought [18, 24, 25, 34]. We next examined whether *BiP* overexpression in Arabidopsis also conferred tolerance to drought. For the drought treatment, water was withheld from 5–week–old plants for 20 days and the pictures and samples were taken at the time points, as indicated in Fig. 8. A water stress tolerant phenotype was clearly developed by the transgenic lines overexpressing *soyBiPD* (Fig. 8a), *AtBiP1* and *AtBiP2* (Additional file 8). This phenotype was typical of tobacco and soybean *BiP*–overexpressing lines, such as maintenance of leaf turgidity (Fig. 8a and Additional file 8), higher relative water content (Fig. 8b) under a water deficit regime and attenuation of drought–mediated induction of the *AtNRP1* gene (Fig. 8c). These results indicate that conserved regulatory mechanisms account for the BiP modulation of drought tolerance and NRP–mediated cell death signaling in different plant species.

## Discussion

### The components of the DCD/NRP–mediated cell death signaling is structurally conserved in the plant kingdom

DCD/NRP–mediated cell death pathway connects osmotic and ER stress on *NRP* genes to activate a cell death program. This pathway was originally identified in soybean [23], and circumstantial evidence indicates that it also operates in tobacco [24]. In this investigation, we extended the characterization of the cell death pathway by demonstrating that it is also conserved in other plant species. Blast searches of the soybean NRPs, GmNAC30, GmNAC81 and VPE sequences against 10 plant genomes, including 7 dicotyledonous species and 3 monocotyledonous species revealed that the components of the NRP–mediated cell death signaling are widely distributed in the plant kingdom. Plylogenetic analysis identified homologs in all plant species, based on the criteria of sequence similarity and clustering in the same soybean genes–derived clades. Remarkably, all analyzed plant genomes harbor homologs of all components of the NRPs/GmNAC81/GmNAC30/VPE signaling module, which share significant structural and sequence similarities with their soybean counterparts.

All homolgs were further examined for conservation in structural configuration (conserved motifs and domains). The homologs of DCD/NRPs from the other plant species share high sequence conservation with GmNRP–A–and GmNRP–B–deduced protein sequences and they cluster together as subgroup I of the DCD domain–containing protein family [29]. Members of this subgroup contain a highly conserved DCD domain at C–terminus and a more divergent N–rich domain at the N–terminus and they are plant–specific proteins. The downstream components of the DCD/NRP–mediated cell death signaling, GmNAC81 and GmNAC30, belong to a plant–specific family of transcriptional factors, the NAC (NAM, ATAF1/2 and CUC2) domain–containing superfamily of transcription factors. While *GmNAC81* is a member of the subgroup TERN (Tobacco elicitor–responsive gene–encoding NAC domain protein), which is induced by elicitors of the pathogen response [35, 36], *GmNAC30* is placed within the ANAC002/ATAF1 (At1G01720) sub–family, which is induced by abiotic stress conditions [36]. All GmNAC81 and GmNAC30 homologs harbor a NAC conserved domain at the N–terminus and a predicted nuclear localization signal. The high conservation of the components of the ER stress NRP–mediated cell death response in the plant kingdom suggests that this cell death signaling response may be a general ER stress response in plants rather a specific transduction pathway in soybean.

### DCD/NRP–mediated cell death response is functionally conserved in Arabidopsis

We provided several lines of evidence revealing that the NRP–mediated cell death pathway also propagates a stress–induced cell death signal in Arabidopsis with features of a PCD response. First, orthologs of all pathway components are present in the Arabidopsis genome. Among the DCD domain–containing N–rich proteins from Arabidopsis, AtNRP1 and AtNRP2, as designated in this investigation, share high conservation of sequence with GmNRP–A and GmNRP–B from soybean and belong to the same clade in phylogenetic analysis. Likewise, another component of the DCD/NRP–mediated cell death signaling, GmNAC81, shares 62 % of identity with the Arabidopsis protein ANAC036. We also found two orthologs of the plant–specific VPE encoded by the genome of Arabidopsis. Second, similarly to the soybean orthologs, *AtNRP1*, *AtNRP2*, *ANAC036* and *gVPE* are induced by osmotic and ER stress. We did not observe, however, a synergistic induction of the Arabidopsis othologs by a combined treatment of both stresses, as already reported for the soybean cell death pathway components [17, 20]. These differences in the expression profile may be explained by differences in the experimental conditions of the assays. The optimal conditions for Arabidopsis exposition to the combined treatment, which would prevent the osmotic stress inducer PEG to interfere with tunicamycin uptake, have not been established. In soybean, the plants are pre–treated with tunicamycin for six hours, and then PEG is added for an additional ten hours [23]. Third, as expected for components of a cell death signaling, the Arabidopsis DCD/NRP–mediated cell death genes can promote PCD *in planta*. Transient expression of *AtNRP1*, *AtNRP2* and *ANAC036* in tobacco leaves causes leaf yellowing, which was associated with chlorophyll loss in agroinfiltrated leaf sectors that evolved to necrotic lesions and enhanced lipid peroxidation (Fig. 3 and Additional file 4), a phenotype similar to that developed by *GmNRP*–*A*, *GmNRP*–*B* and *GmNAC81* expression. This process of accelerated leaf yellowing induced by expression of DCD/NRP cell death pathway components has been shown to be associated with programmed cell death in soybean [17, 18, 20]. Furthermore, expression of *AtNRP1*, *AtNRP2* and *ANAC036* in tobacco protoplasts promoted extensive DNA fragmentation to similar extent as in the *GmNRP*–*B*–expressing control samples. The extensive cleavage of nuclear DNA is one feature of cell death. Finally, loss of *AtNRP1*, *AtNRP2* function attenuated ER stress–induced cell death, whereas overexpression of AtNRP2 enhances cell death (Fig. 4), confirming that these genes transduce a cell death signal in Arabidopsis.

A functional conservation between *AtNRP1*, *AtNRP2*, *GmNRP*–*A* and *GmNRP*–*B* orthologs was further demonstrated in complementation assays of the hypersensitive phenotype to osmotic stress displayed by the atnrp1 null mutants. Under osmotic conditions, a further enhancement of root growth inhibition is clearly observed in the *atnrp1* line (Fig. 5a). Stable expression of *AtNRP1*, *AtNRP2*, *GmNRP*–*A* or *GmNRP*–*B* in the atnrp1 null mutant rescued the wild–type phenotype and the root growth of transgenic lines under PEG treatment was compared to the untransformed wild–type line. These results indicated that NRPs may be involved in root growth under osmotic stress. Consistent with this hypothesis, overexpression of *BiP*, a negative regulator of *AtNRP1* expression, recapitulates the enhanced phenotype of PEG–mediated root growth inhibition of atnrp1 null alleles (Fig. 5b). Likewise, *BiP* overexpression in soybean also promotes root inhibition under osmotic stress and drought and represses *GmNRP*–*A* and *GmNRP*–*B* expression [24]. The NRP involvement in root growth is not likely associated with a cell death function but rather it may be linked to the pro–survival activity of UPR. Two lines of evidence argue favorably for this hypothesis. First, both GmNARP–A and GmNRP–B promoters harbor putative unfolded protein response elements (UPRE2AT on GmNRP–A promoter and UPRE1AT on GmNRP–B promoter; Additional file 9), and the AtNRP1 promoter contains two putative UPREs (UPRE1A and UPRE2A; Additional file 9), which have the potential to be activated by UPR branches under ER stress (http://plantpan2.itps.ncku.edu.tw) [37]. Second, loss of *IRE1a* and *IRE1b* function causes root inhibition indicating the relevance of an activated UPR in root development [15, 38]. Under stress conditions, root growth requires a functional bZIP60 in addition of IRE1a/IRE1b, implicating the IRE1a/IRE1b–bZIP60 module of UPR as a critical player not only in stress tolerance but also in root growth. However, it remains to be determined whether induction and function of NRPs under osmotic stress are linked to the IRE1a/IRE1b–bZIP60 arm of the UPR.

The components of the DCD/NRP cell death signaling pathway have been identified in soybean mostly as a result of biochemical and forward genetic approaches and their hierarchical order in the pathway has been assigned based on expression and kinetic studies [20, 21, 23]. In the Arabidopsis system, however, the characterization of the atnrp1 null alleles confirmed that AtNRP1 is upstream of GmNAC36 and *AtNRP2* expression depends on *AtNRP1* function. In fact, loss of *AtNRP1* function prevents osmotic and ER stress–mediated induction of *AtNRP2* and the other downstream components of the pathway, *ANAC036* and *VPE*. Consistent with this interpretation, AtNRP1 and AtNRP2 transactivate the AtNRP2 promoter, but not the AtNRP1 promoter. As a downstream component, the transcriptional factor ANAC036 does not feedback regulate the *AtNRP1* and *AtNRP2* upstream components. Recently, we demonstrated that the ANAC036 soybean homolog, GmNAC81, interacts with another member of the NAC family, GmNAC30, to activate fully the expression of the downstream component *VPE*. It would be very interesting to investigate whether ANAC036 would require another member of the NAC family for full transactivation of the VPE promoter, as for GmNAC81 [21].

### *BiP* overexpression promotes water stress tolerance and modulates DCD/NRP–mediated cell death response in Arabidopsis

BiP has been demonstrated to protect plants against abiotic and biotic stress conditions through gene expression modulation and posttranslational regulation of secretory proteins [17, 18, 24, 25, 34, 39–41]. As a molecular chaperone, BiP attenuates the ER stress response [17, 40] by regulating the activity of the UPR transducer bZiP28 [42]. In the absence of stress, plant BiP is bound to Arabidopsis ATF6-like bZIP28, which remains in the ER membrane [43]. Upon ER stress, BiP dissociates from bZIP28, allowing it to be translocated to the Golgi, where it is proteolytically cleaved by S1P/S2P and released from the membrane to be redirected to the nucleus [43, 44]. In addition to controlling UPR, plant BiP displays protective functions under distinct stress conditions, including (i) the attenuation of ER stress [34, 40], (ii) the promotion of drought tolerance in transgenic soybean (*Glycine max*) and tobacco (*Nicotiana tabacum*) plants [24, 34], (iii) the activation of plant innate immunity [45] and (iv) the attenuation of ER stress–and osmotic stress–induced cell death in soybean [18]. The BiP protective properties under a water deficit regime are linked to its capacity to control the activity and expression of the cell death NRP/GmNAC81/GmNAC31/VPE signaling module [18, 25]. Here, we demonstrated that the NRP–mediated cell death signaling is also controlled negatively by BiP in *Arabidopsis thaliana*. Overexpression of the *soyBiPD* and Aradidopsis *BiP1*/*BiP2* genes attenuated the osmotic and ER stress induction of the cell death signaling genes *AtNRP1*, *AtNRP2*, *ANAC036* and *VPE*. The BiP attenuation of cell death responses has been associated with BiP–mediated increases in water stress tolerance in soybean and tobacco [24, 34]. Accordingly, in Arabidopsis, *BiP*–overexpressing lines were more tolerant to drought. The *BiP*–overexpressing lines maintained leaf turgidity under dehydration conditions caused by drought stress, a typical phenotype mediated by *BiP* overexpression in soybean and tobacco [24, 34]. Our current results revealed the conservation for a BiP negative regulation of NRP–mediated cell death response in the plant kingdom and implicated BiP as potential target for engineering water stress tolerance in other crops.

## Conclusion

As plants are exposed to environmental changes and extreme conditions, they have developed coordinated and integrated mechanisms, which respond to these injuries and are immediately activated upon stresses. One such important mechanism is the DCD/NRP–mediated cell death response, which was first identified in soybean and is induced by multiple stresses. Our results have demonstrated that this cell death signaling response may be a general stress response in plants rather a specific transduction pathway in soybean. This interpretation was based on *in silico* analysis of pathway homologs in the plant kingdom, which was complemented with functional studies of the cell death–signaling module in Arabidopsis. In addition to being induced by ER and osmotic stress, the Arabidopsis components of the signaling module promoted extensive DNA fragmentation in tobacco protoplasts and induced leaf yellowing, chlorophyll loss and lipid peroxidation *in planta*, a reminiscent PCD phenotype induced by the soybean orthologs. Furthermore, loss of *AtNRP1*, *AtNRP2* function attenuated ER stress–induced cell death and overexpression of AtNRP2 enhanced cell death (Fig. 4), confirming that these genes transduce a cell death signal in Arabidopsis. By using reverse genetics, the Arabidopsis orthologs were genetically linked to the cell death response in sequential order, conclusively defining the plant–specific NRP/ANAC36/VPE signaling module. Like in soybean, BiP attenuated the propagation of the stress–induced cell death signal in Arabidopsis by modulating the expression and activity of the signaling module and hence conferred tolerance to drought. Collectively, our results indicated that the components of the cell death–signaling module are structurally conserved in the plant kingdom and function in Arabidopsis with conserved regulatory mechanism. The demonstration of regulatory and functional conservation of the stress–induced NRP–mediated cell death signaling in plants revealed a broad–spectrum potential for targeting the BiP–mediated increases in drought tolerance in different crops.

## Methods

### Phylogenetic analyses

The previously characterized soybean genes of the NRP–mediated cell death signaling, *GmNRP*–*A*, *GmNRP*–*B*, *GmNAC81*, *GmNAC30* and *VPE*, were used as prototypes for Blast searching orthologs against the genomes of Arabidopsis thaliana, Carica papaya, Citrus sinensis, Cucumis sativis, Glycine max, Manihot esculenta, Phaseolus vulgaris, Solanum lycopersicum, Solanum tuberosum, Triticum aestivum, Oryza sativa and Zea mays. For each signaling module component, we selected the five more related sequences of each plant species to construct phylogenetic trees using Bayesian inference. The amino acid sequences of orthologous genes were recovered from TAIR (http://arabidopsis.org/) and Phytozome v10.3 databases. For phylogenetic analyses, the amino acid sequences were aligned using MUSCLE [46]. Phylogenetic trees were constructed using Bayesian inference performed with MrBayes v3.2.2 [47] with mixed amino acid substitution model (Blosum). The analyses were carried out running 20.000.000 generations and excluding the first 5.000.000 generations as burn–in. The trees were visualized with Figtree v1.4 software (http://tree.bio.ed.ac.uk/software/figtree/).

### Plasmid Construction

For transient expression in protoplasts and *N. benthamiana* leaves and for Arabidopsis transformation *AtNRP1*, *AtNRP2*, *ANAC036*, *AtBiP1*, *AtBiP2*, *gVPE* and *soyBiPD* were amplified from Arabidopsis and soybean cDNA using specific primers (AtNRP1 Fwd and AtNRP1 Rvs; AtNRP2 Fwd and AtNRP2 Rvs; ANAC036 Fwd and ANAC036 Rvs, BiP1 Fwd and BiP1 Rvs, BiPD Fwd and BiPD Rvs, VPE–gFwd and VPE–g Rvs) (Additional file 10) and inserted by recombination into the entry vectors pDONR201 and pDONR221 (Invitrogen) to yield pDONR201Ns–AtNRP1, pDONR201Ns–AtNRP2, pDONR201–ANAC036, pDONR221BiPD, pDONR201BiP1, pDONR201BiP2 and pDONR201VPE–g. These genes were then transferred from the entry vectors to different expression vector (pK7WG2, pEarleygate100 and pEarleygate 104) by recombination using the enzyme LR clonase (Invitrogen) to generate the clones described here: p35S: AtNRP1, p35S: AtNRP2, p35S: ANAC036, p35S: BiPD, p35S: BiP1, p35S: BiP2, p35S: VPE–g. For the transient expression in the protoplasts and tobacco, AtNRP1, AtNRP2, ANAC036, BiPD, BiP1 and BiP2 were fused to YFP (pYFP–AtNRP1,pYFP–AtNRP2, pYFP–ANAC036, pYFP–BiPD, pYFP–BiP1 and pYFP–BiP2) or to GFP (gVPE–GFP) and placed under the control of the 35S promoter in the respective binary vector for plant transformation.

For Arabidopsis transformation, AtBiP1 and AtBiP2 cDNAs were amplified from Arabidopsis cDNA and inserted by recombination into the entry vector pDONR201 (Invitrogen) to yield pDON–BiP1 and pDONBiP2. These cDNAS were transferred from the entry vector to pK7FWG2 to generate pK7F–BiP1 and pK7F–BiP2, which harbor the respective BiP coding region fused to N–terminus of GFP. The BiP–GFP fusions were them amplified using reverse oligonucleotides with an extension of the HDEL–encoding sequence and reinserted into pDONR201. Then, BiP–GFP–HDEL encoding sequences were transferred by recombination to pK7WG2 generating pK7–BiP1 and pK7–BiP2, which harbor BiP1–GFP–HDEL or BiP2–GFP–HDEL under the control of the 35S promoter, respectively.

To transform Arabidopsis with *soyBiPD*, we used the previously described clone pUFVBiPS [34], in which the soyBiPD cDNA, which harbors the native HDEL sequence at the C–terminus, was placed under the control of the 35S promoter and the 3′*nos* polyadenylation signal in the plant transformation binary vector pBI121. The independently transformed lines BiPDox T07, BiPox T10, BiPDox T13 and BiPox T27 were selected for further analysis.

### Plant Growth and transformation

Arabidopsis thaliana ecotype Columbia (Col–0) was used as the wild–type control. The Arabidopsis T–DNA mutant *atnrp1* (SALK_041306, insertion in exon) were obtained from the Arabidopsis Biological Resource Center. The primers used for genotyping are listed in Additional file 10. Surface–sterilized seeds were plated directly onto square Petri dishes containing 1/2 Linsmaier and Skoog (LS) medium, and 0.7 % agar. For normal growth conditions, plants were grown at 21 °C under a 16 h light/8 h dark cycle. *Agrobacterium*–mediated transformation was performed using the floral–dip method [48]. *Agrobacterium* strain GV3101 was used in all transformation experiments.

### Immunoblot analysis

Total protein was extracted from leaves of untransformed or transformed Arabidopsis plants as previously described by Cascardo et al. [49]. The isolation of the microsomal fraction from Arabidopsis leaves was performed as described by Pirovani et al. [50]. SDS–PAGE was carried out and the proteins were transferred from 10 % SDS–polyacrylamide gels to nitrocellulose membranes by electroblotting. Immunoblot analyses were performed using polyclonal BiP antibodies prepared against an E. coli produced BiP carboxy domain (anti–carboxy BiP) derived from soyBiPD [51], at a 1:1000 dilution and a goat anti–rabbit IgG alkaline phosphatase conjugate (Sigma) at a 1:5000 dilution. Alkaline phosphatase activity was assayed using 5–bromo–4–chloro–3–indolyl phosphate (Sigma.) and p–nitro blue tetrazolium (Sigma).

### Real–time RT–PCR Analysis

For quantitative RT–PCR, total RNA was extracted from frozen leaves or cells with TRIzol (Invitrogen) according to the instructions from the manufacturer. The RNA was treated with 2 units of RNase–free DNase (Promega) and further purified through RNeasy Mini kit (Qiagen) columns. First–strand cDNA was synthesized from 4 μg of total RNA using oligo–dT (18) and Transcriptase Reversa M–MLV (Invitrogen), according to the manufacturer’s instructions. Real–time RT–PCR reactions were performed on an ABI7500 instrument (Applied Biosystems), using SYBR_ Green PCR Master Mix (Applied Biosystems). The amplification reactions were performed as follows: 2 min at 50 °C, 10 min at 95 °C, and 40 cycles of 94 °C for 15 s and 60 °C for 1 min. To confirm quality and primer specificity, we verified the size of amplification products after electrophoresis through a 1.5 % agarose gel, and analyzed the Tm (melting temperature) of amplification products in a dissociation curve, performed by the ABI7500 instrument. The used primers are listed in Additional file 10. For quantitation of gene expression in Arabidopsis seedlings, we used actin 2 (At3g18780) [52] or UBQ5 (At3g62250) [53] as the endogenous control gene for data normalization in real–time RT–PCR analysis. Fold variation, which is based on the comparison of the target gene expression (normalized to the endogenous control) between experimental and control samples, was quantified using the comparative Ct method 2^–(∆CtTreatment–∆CtControl)^. The absolute gene expression was quantified using the 2^–∆CT^ method, and values were normalized to the endogenous control.

### PEG and tunicamycin treatment

In the plate system, tunicamycin (TUN; Sigma, dissolved in DMSO) or PEG (MW 8000, Sigma) was directly added to 1/2 LS medium containing 0.7 % agar, at the concentrations indicated. Seeds were directly germinated in TUN–containing medium or PEG–containing medium for induction of ER stress tolerance and osmotic stress, respectively. To harvest tissue for gene expression analysis under osmotic or ER stress, the seeds were germinated in 1/2 LS medium for 2 weeks and then transferred to TUN–containing medium or PEG–containing medium.

### Drought tolerance

Drought tolerance assay was performed on 5–week–old seedlings. After germination on 1/2 LS plates, 7–d–old seedlings of transgenic lines were planted in sieve–like rectangular plates (3 cm deep) filled with a mixed soil that had been well watered. The seedlings were cultured in a greenhouse (22 °C, 70 % humidity, 120 mmol. m22.s21, 12 h light/12 h dark cycle) without watering for 20 days.

### Determination of chlorophyll content and lipid peroxidation

Total chlorophyll content was determined spectrophotometrically at 663 nm and 646 nm after quantitative extraction from individual leaves with 80 % (v/v) acetone as described by Lichtenthaler [54]. The extent of lipid peroxidation in leaves was estimated by measuring the amount of MDA, a decomposition product of the oxidation of polyunsaturated fatty acids. The malondialdehyde (MDA) content was determined by the reaction of thiobarbituric acid (TBA) as described by Hodges et al. [55].

### Transient Expression in tobacco Protoplasts

Protoplasts were prepared from soybean suspension cells, as essentially described by Costa et al. [17]. The protoplasts were isolated from tobacco leaves by digestion for 3 h, under agitation at 40 rpm, with 0.5 % (w/v) cellulase, 0,5 % (w/v) macerozyme R–10, 0.1 % (w/v) pectolyase Y23, 0.6 M mannitol, 20 mM MES, pH 5.5. The extent of digestion was monitored by examining the cells microscopically at each 30–min interval. After filtration through nylon mesh of 65 μm, protoplasts were recovered by centrifugation, resuspended in 2 mL of 0.6 M mannitol, 20 mM MES, pH 5.5, separated by centrifugation in a sucrose gradient (20 % (w/v) sucrose, 0.6 M mannitol, 20 mM MES, pH 5.5), and diluted into 2 mL of electroporation buffer (25 mM Hepes–KOH, pH 7.2, 10 mM KCl, 15 mM MgCl2, 0.6 M mannitol). Transient expression assays were performed by electroporation (250 V, 250 μF) of 10 μg of expression cassette DNA, and 30 μg of sheared salmon sperm DNA into 2 × 10^5^–5 × 10^6^ protoplasts in a final volume of 0.8 mL. Protoplasts were diluted into 8 mL of MS medium supplemented with 0.2 mg/mL 2,4–dichlorophenoxyacetic acid and 0.6 M mannitol, pH 5.5. After 36 h of incubation in the dark, the protoplasts were washed with 0.6 M mannitol, 20 mM MES, pH 5.5 and processed.

### *in Situ* Labeling of DNA Fragmentation (TUNEL)

Free 3′–OH in the DNA was labeled by the terminal deoxynucleotidyl transferase–mediated dUTP nick end labeling (TUNEL) assay using the ApoAlert DNA Fragmentation Assay kit (Clontech), as instructed by the manufacturer. Samples were observed with a Zeiss LSM 410 inverted confocal laser scanning microscope fitted with the configuration: excitation at 488 nm and emission at 515 nm.

### Evans blue staining

Seedlings were treated with Tm (5 μg/mL) for 24 h. Tunicamycin–and DMSO–treated seedlings were stained with 2 % (w/v) Evans blue for 3 min and then extensively washed with water.

### Glucuronidase Transactivation Assays

A 2,000–bp fragment of the 5′ flanking sequences of the *AtNRP1*, *AtNRP2* genes, relative to the translational initiation codon, were amplified from Arabidopsis DNA with the primers listed in Additional file 10. The amplified fragments were cloned into the pDONR221 entry vector (Invitrogen) and then transferred to pGWB203 by recombination with LR clonase to yield pAtNRP1pro: GUS (glucuronidase) and pAtNRP2pro: GUS. Tobacco leaves were agroinfiltrated with eacha promoter construct in combination with YFP–AtNRP1, YFP–AtNRP2 or YFP–ANAC036. After three days, the protein extraction and fluorometric assays for GUS activity were performed with methylumbelliferone as a standard. For the standard assay, the leaves were ground in 0.5 mL GUS assay buffer [100 mM NaH2PO4 · H2O (pH 7.0), 10 mM EDTA, 0.1 % (w/v) sarkosyl, and 0.1 % (v/v) Triton X–100], and 25 μL of this extract were mixed with 25 μL GUS assay buffer containing 2 mM fluorescent4–methylumbelliferyl β–D glucuronide as a substrate. The mixture was incubated at 37 °C in the dark for 30 min, and the GUS activity was measured using a Lector Multi–Mode Microplate Reader Synergy HT (BioTek). The total protein concentration was determined by the Bradford method. The experiments were repeated three times with similar results.

## Abbreviations

BiP, binding protein; DCD, developmental and cell death domain; ER, endoplasmic reticulum; GmNAC81, Glycine max NAC81 transcription factor; NRP, asparagine (N)–rich proteins; PCD, programmed cell death; TF, transcription factor; VPE, vacuolar processing enzyme

## Additional files

Additional file 1: Phylogenetic analysis of *GmNAC81*–like genes. The amino acid sequences of GmNAC81–like proteins were recovered from TAIR (http://arabidopsis.org/) and Phytozome v10.3 databases and aligned using MUSCLE. Phylogenetic trees were constructed as described in Fig. 1. (TIF 2645 kb) Additional file 2: Plylogentic analysis of *GmNAC30*–related genes. The amino acid sequences of GmNAC30–like proteins were recovered from TAIR (http://arabidopsis.org/) and Phytozome v10.3 databases and aligned using MUSCLE. Phylogenetic trees were constructed as described in Fig. 1. (TIF 2730 kb) Additional file 3: Phylogenetic analysis of *VPE*–like genes. The amino acid sequences of VPE–like proteins were recovered from TAIR (http://arabidopsis.org/) and Phytozome v10.3 databases and aligned using MUSCLE. Phylogenetic trees were constructed as described in Fig. 1. (TIF 2373 kb) Additional file 4: Arabidopsis NRPs and ANAC036 cause cell death *in planta*. Leaves from 3 weeks–old *N. benthamiana* were infiltrated with agrobacterium cells transformed with p35S: AtNRP1 (A), p35S: AtNRP2 (B), p35S: ANAC036 (C) p35S: NRP–A (D), p35S: NRP–B (E) expression vectors or with control binary expression vectors harboring an unrelated gene from soybean (F). Pictures were taken 6 days after infiltration. (G) Chlorophyll loss induced by AtNRP1, AtNRP2, ANAC036, NRP–A and NRP–B expression. Total chlorophyll, chlorophyll a and b were determined from the leaf sectors agroinfiltrated with the described DNA constructions. Error bars indicate the 95 % confidence interval based on a t–test (*p* < 0,05, *n* = 3). (TIF 1835 kb) Additional file 5: Expression analysis of transgenes and knockout genes. a, b, c, d Expression analysis of transgenes transiently expressed in agroinfiltrated *N. benthamiana* leaves. Total RNA was isolated from agroinfiltrated leaves and the transcript levels of selected genes, as indicated, were quantified by qRT–PCR. Gene expression was calculated using the 2^-ΔCt^ method and *actin* as endogenous control. Values represent mean ± S.D. from three replicates. e Accumulation of AtNRP1 transcript in atnrp1 knockout line. RT–PCR was performed on leaf RNA samples from Col–0 and atnrp1 plants with gene–specific primers for *AtNRP1* or *ERD15*, as a negative control. (TIF 484 kb) Additional file 6: Soybean BiPD accumulation in independently transformed transgenic Arabidopsis lines. a Coomassie–stained protein gel and (b) immunoblot of whole cell protein extracts and microsomal fractions of Arabidopsis leaves. Equal amounts of whole cell protein extract (20 μg) and microsomal fraction (20 μg) from leaves of Col–0 (wild type) and transgenic lines (T07, T10, T13, T23) were separated by SDS–PAGE and either stained with Coomassie Brilliant Blue R250 (a) or transferred to nitrocellulose and immunoblotted with anti–carboxy BiP serum (b). c Soybean BiPD is correctly localized in the microsomal fraction. SoyBiPD accumulation was monitored as in a, but in addition to the total protein (TP) and the microsomal fraction (MF), we included in the blot the cytosolic fraction (CF), which was probed with an anti–cytosolic UGPase serum to certify that BiP accumulation was restricted to the microsomal fraction of transgenic lines. (TIF 3471 kb) Additional file 7: Arabidopsis BiP1 and BiP2 modulate the NRP–mediated cell death signaling and cause tolerance to drought. a Enhanced accumulation of Arabidopsis BiP1 or BiP2 transcripts in transgenic lines. Total RNA was isolated from wild–type leaves, *BiP1*–overexpressing (BiP1ox) and *BiP2*–overexpressing (BiP2ox) transgenic leaves and BIP transcript levels were determined by quantitative RT–PCR. The relative expression was quantified using the 2^–ΔΔCt^ method and *UBQ5* as an endogenous control. The values are relative to the control treatment (wild–type), and error bars indicate the 95 % confidence interval based on a t–test (*p* < 0,05, *n* = 3). b AtBiP1–GFP–HDEL and AtBiP2–GFP–HDEL accumulation in Arabidopsis transgenic lines. Total protein from Col–0 and transgenic lines were separated in SDS–PAGE and immunoblotted with an anti–soyBiPD serum, which recognizes both the endogenous BiP1 and BiP2 (lower bands) and the fusion proteins (upper bands). In the lower blot, the recombinant proteins were probed with an anti–GFP serum to recognize specifically BiP–GFP fusion proteins. c The BiP–GFL–HDEL fusion protein localized in the microsomal fraction in transgenic lines. Equal amounts of whole cell protein extract (TF), cytosolic fraction (CF) and microsomal fraction (MF) from seedlings of Col–0 (upper blot) were separated by SDS–PAGE, immunoblotted with an anti–BiP serum and reprobed with an anti–UGPase, as a cytosolic marker. Asterisks indicate anti–UGPase cross–reacting proteins. In the lower blot, the BiP–GFP–HDEL fusion was probed with an anti–GFP serum. d and e Arabidodpis *BiP* overexpression attenuates the tunicamycin and PEG induction of DCD/NRP–mediated cell death signaling genes. Total RNA was isolated from 15 days–old Arabidopsis plants treated with PEG (10 % w/v) and Tunicamycin (2,5 μg/mL) for 2 h, 6 h, 12 h and 24 h. H_2_O was used as control for PEG and DMSO for Tunicamycin. The transcript levels of selected genes were quantified by qRT–PCR. Gene expression was calculated using the 2^-ΔΔCt^ method and *UBQ5* as endogenous control. cDNAs were obtained from four biological replicates and validated individually. The error bars indicate the 95 % confidence interval based on a t–test (*p* < 0,05, *n* = 4). (TIF 969 kb) Additional file 8: Enhanced accumulation of Arabidopsis BiP confers tolerance to drought. Arabidopsis plants, genotypes Col–0, BiPDox, BiP1ox and BiP2ox lines were grown in soil and water stress was induced by withholding irrigation for 20 days. Photography was taken at the indicated days after withholding irrigation. (TIF 6777 kb) Additional file 9: Unfolded protein response elements (UPRE) on the promoter of development cell death (DCD)/N–rich proteins (NRPs). (DOCX 58 kb) Additional file 10: Primers used for cloning and quantitative RT–PCR. (DOCX 126 kb)
