# Supplementary figures and images for: Functional and regulatory conservation of the soybean ER stress-induced DCD/NRP-mediated cell death signaling in plants
Source: BMC Plant Biol. 2016 Jul 12;16:156. doi: 10.1186/s12870-016-0843-z (PMC4943007; doi:10.1186/s12870-016-0843-z)

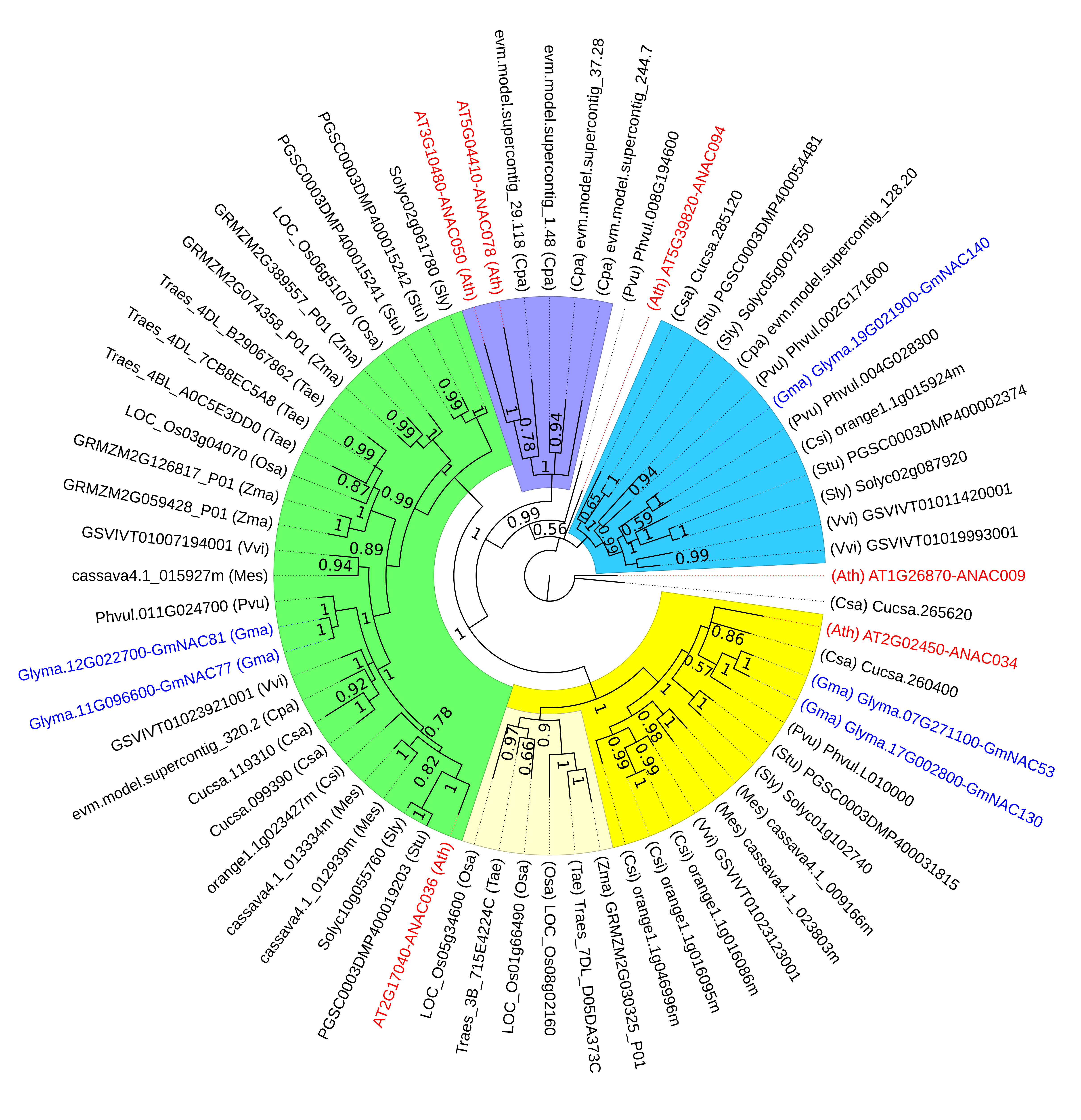

Supplement: Additional file 1: — Phylogenetic analysis of GmNAC81–like genes. The amino acid sequences of GmNAC81–like proteins were recovered from TAIR (http://arabidopsis.org/) and Phytozome v10.3 databases and aligned using MUSCLE. Phylogenetic trees were constructed as described in Fig. 1. (TIF 2645 kb) [file 12870_2016_843_MOESM1_ESM.tif]

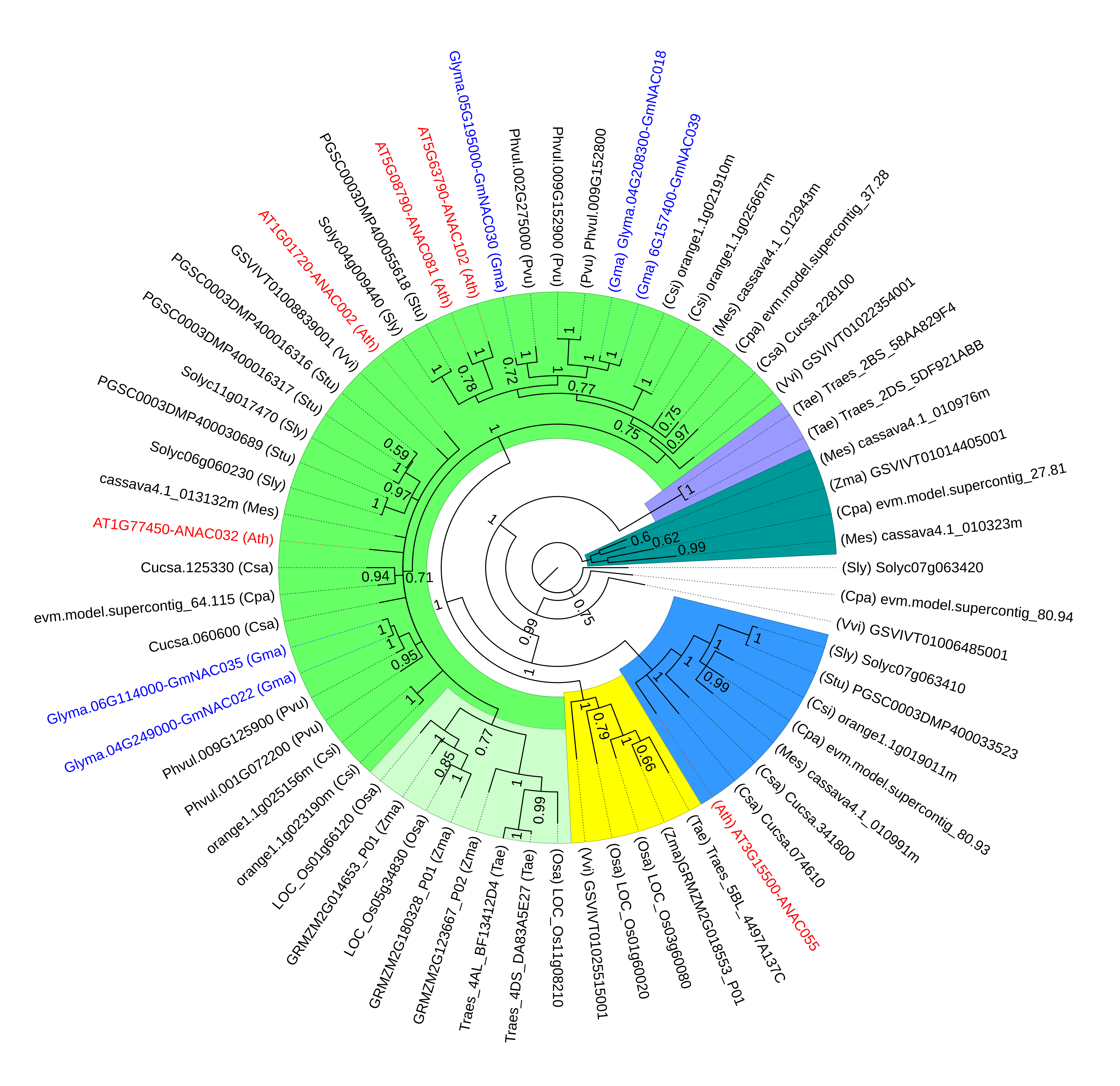

Supplement: Additional file 2: — Plylogentic analysis of GmNAC30–related genes. The amino acid sequences of GmNAC30–like proteins were recovered from TAIR (http://arabidopsis.org/) and Phytozome v10.3 databases and aligned using MUSCLE. Phylogenetic trees were constructed as described in Fig. 1. (TIF 2730 kb) [file 12870_2016_843_MOESM2_ESM.tif]

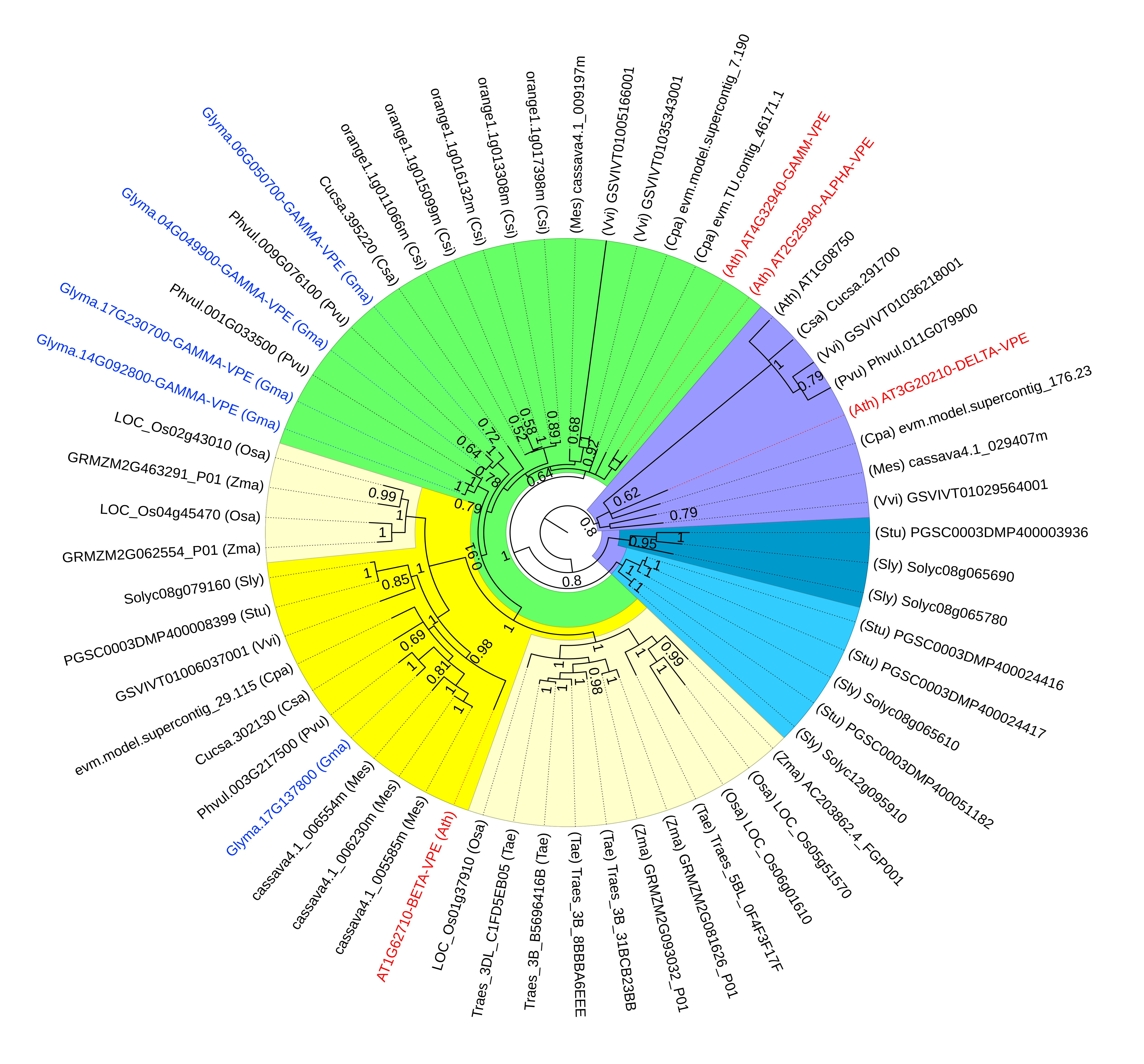

Supplement: Additional file 3: — Phylogenetic analysis of VPE–like genes. The amino acid sequences of VPE–like proteins were recovered from TAIR (http://arabidopsis.org/) and Phytozome v10.3 databases and aligned using MUSCLE. Phylogenetic trees were constructed as described in Fig. 1. (TIF 2373 kb) [file 12870_2016_843_MOESM3_ESM.tif]

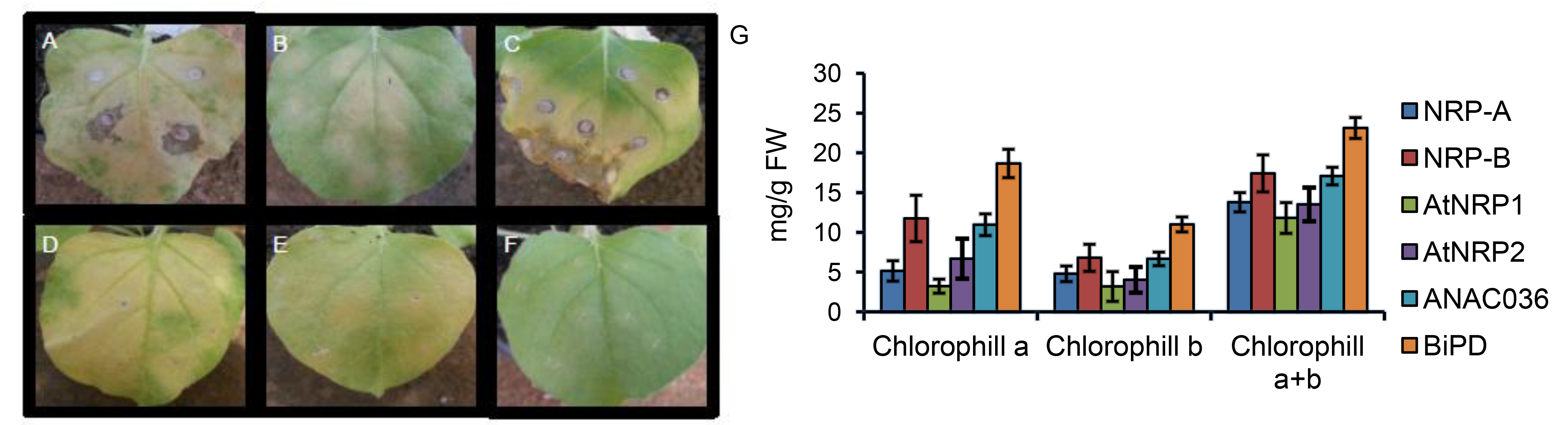

Supplement: Additional file 4: — Arabidopsis NRPs and ANAC036 cause cell death in planta. Leaves from 3 weeks–old N. benthamiana were infiltrated with agrobacterium cells transformed with p35S: AtNRP1 (A), p35S: AtNRP2 (B), p35S: ANAC036 (C) p35S: NRP–A (D), p35S: NRP–B (E) expression vectors or with control binary expression vectors harboring an unrelated gene from soybean (F). Pictures were taken 6 days after infiltration. (G) Chlorophyll loss induced by AtNRP1, AtNRP2, ANAC036, NRP–A and NRP–B expression. Total chlorophyll, chlorophyll a and b were determined from the leaf sectors agroinfiltrated with the described DNA constructions. Error bars indicate the 95 % confidence interval based on a t–test (p < 0,05, n = 3). (TIF 1835 kb) [file 12870_2016_843_MOESM4_ESM.tif]

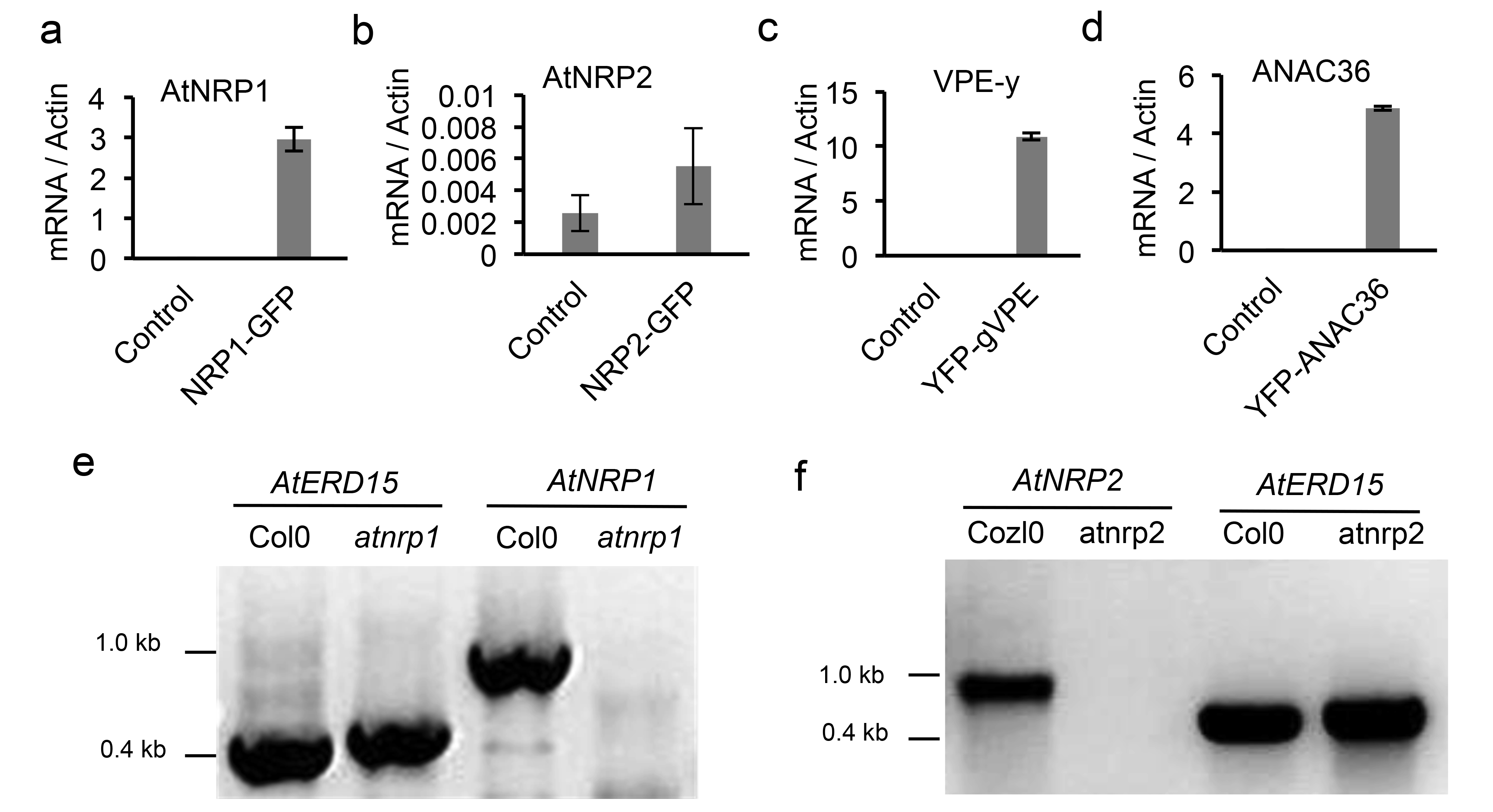

Supplement: Additional file 5: — Expression analysis of transgenes and knockout genes. a, b, c, d Expression analysis of transgenes transiently expressed in agroinfiltrated N. benthamiana leaves. Total RNA was isolated from agroinfiltrated leaves and the transcript levels of selected genes, as indicated, were quantified by qRT–PCR. Gene expression was calculated using the 2-ΔCt method and actin as endogenous control. Values represent mean ± S.D. from three replicates. e Accumulation of AtNRP1 transcript in atnrp1 knockout line. RT–PCR was performed on leaf RNA samples from Col–0 and atnrp1 plants with gene–specific primers for AtNRP1 or ERD15, as a negative control. (TIF 484 kb) [file 12870_2016_843_MOESM5_ESM.tif]

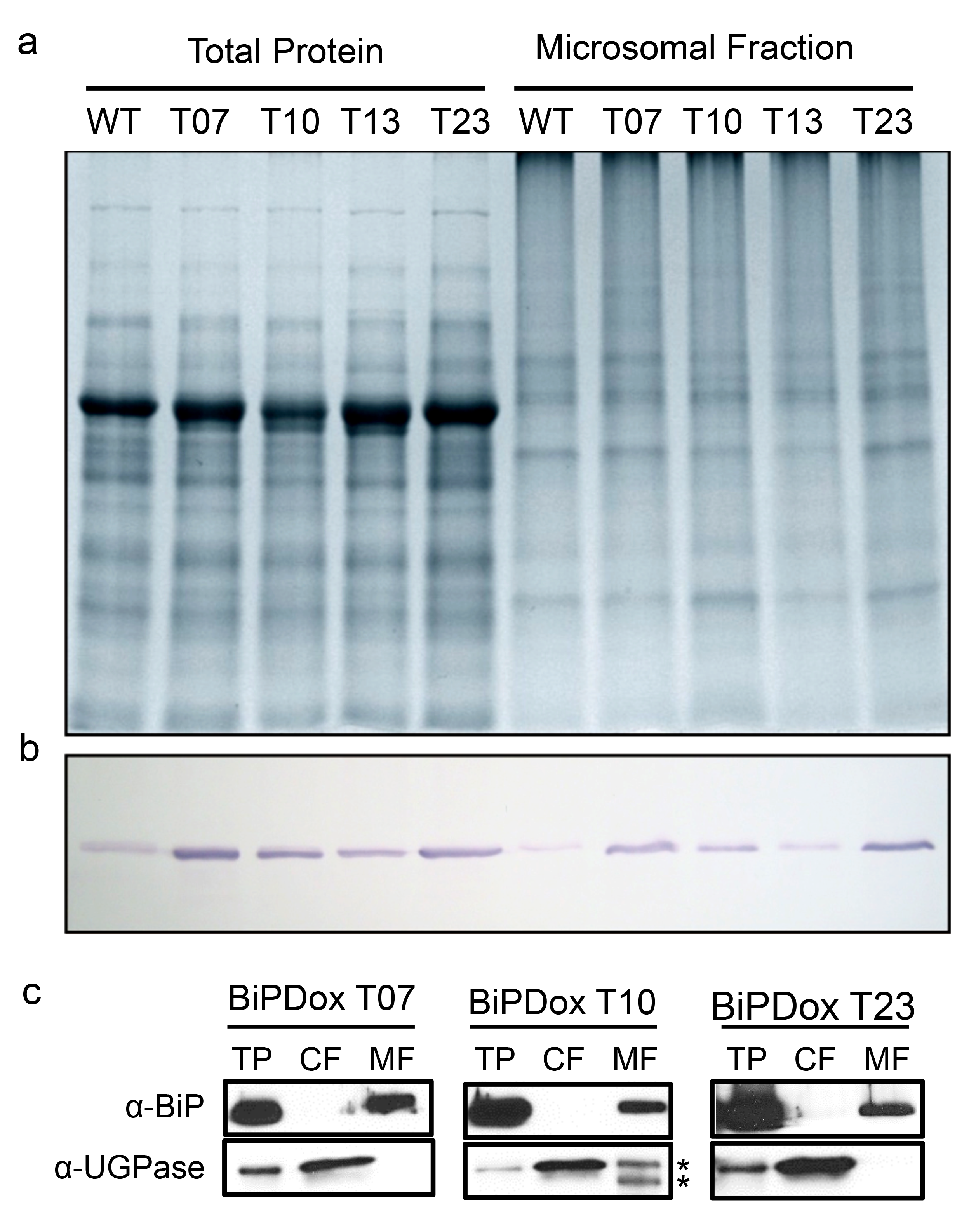

Supplement: Additional file 6: — Soybean BiPD accumulation in independently transformed transgenic Arabidopsis lines. a Coomassie–stained protein gel and (b) immunoblot of whole cell protein extracts and microsomal fractions of Arabidopsis leaves. Equal amounts of whole cell protein extract (20 μg) and microsomal fraction (20 μg) from leaves of Col–0 (wild type) and transgenic lines (T07, T10, T13, T23) were separated by SDS–PAGE and either stained with Coomassie Brilliant Blue R250 (a) or transferred to nitrocellulose and immunoblotted with anti–carboxy BiP serum (b). c Soybean BiPD is correctly localized in the microsomal fraction. SoyBiPD accumulation was monitored as in a, but in addition to the total protein (TP) and the microsomal fraction (MF), we included in the blot the cytosolic fraction (CF), which was probed with an anti–cytosolic UGPase serum to certify that BiP accumulation was restricted to the microsomal fraction of transgenic lines. (TIF 3471 kb) [file 12870_2016_843_MOESM6_ESM.tif]

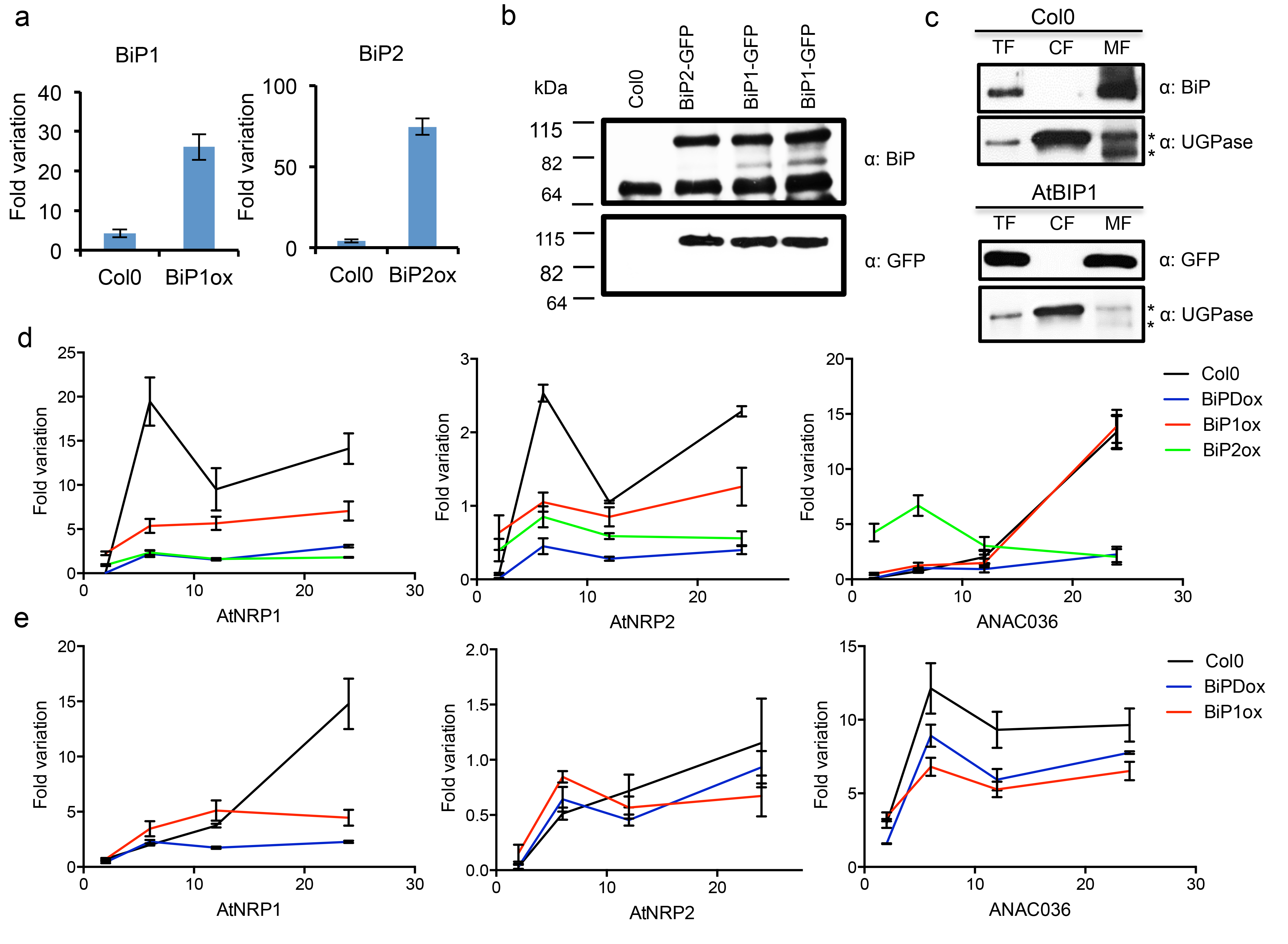

Supplement: Additional file 7: — Arabidopsis BiP1 and BiP2 modulate the NRP–mediated cell death signaling and cause tolerance to drought. a Enhanced accumulation of Arabidopsis BiP1 or BiP2 transcripts in transgenic lines. Total RNA was isolated from wild–type leaves, BiP1–overexpressing (BiP1ox) and BiP2–overexpressing (BiP2ox) transgenic leaves and BIP transcript levels were determined by quantitative RT–PCR. The relative expression was quantified using the 2–ΔΔCt method and UBQ5 as an endogenous control. The values are relative to the control treatment (wild–type), and error bars indicate the 95 % confidence interval based on a t–test (p < 0,05, n = 3). b AtBiP1–GFP–HDEL and AtBiP2–GFP–HDEL accumulation in Arabidopsis transgenic lines. Total protein from Col–0 and transgenic lines were separated in SDS–PAGE and immunoblotted with an anti–soyBiPD serum, which recognizes both the endogenous BiP1 and BiP2 (lower bands) and the fusion proteins (upper bands). In the lower blot, the recombinant proteins were probed with an anti–GFP serum to recognize specifically BiP–GFP fusion proteins. c The BiP–GFL–HDEL fusion protein localized in the microsomal fraction in transgenic lines. Equal amounts of whole cell protein extract (TF), cytosolic fraction (CF) and microsomal fraction (MF) from seedlings of Col–0 (upper blot) were separated by SDS–PAGE, immunoblotted with an anti–BiP serum and reprobed with an anti–UGPase, as a cytosolic marker. Asterisks indicate anti–UGPase cross–reacting proteins. In the lower blot, the BiP–GFP–HDEL fusion was probed with an anti–GFP serum. d and e Arabidodpis BiP overexpression attenuates the tunicamycin and PEG induction of DCD/NRP–mediated cell death signaling genes. Total RNA was isolated from 15 days–old Arabidopsis plants treated with PEG (10 % w/v) and Tunicamycin (2,5 μg/mL) for 2 h, 6 h, 12 h and 24 h. H2O was used as control for PEG and DMSO for Tunicamycin. The transcript levels of selected genes were quantified by qRT–PCR. Gene expression was calculated using the [file 12870_2016_843_MOESM7_ESM.tif]

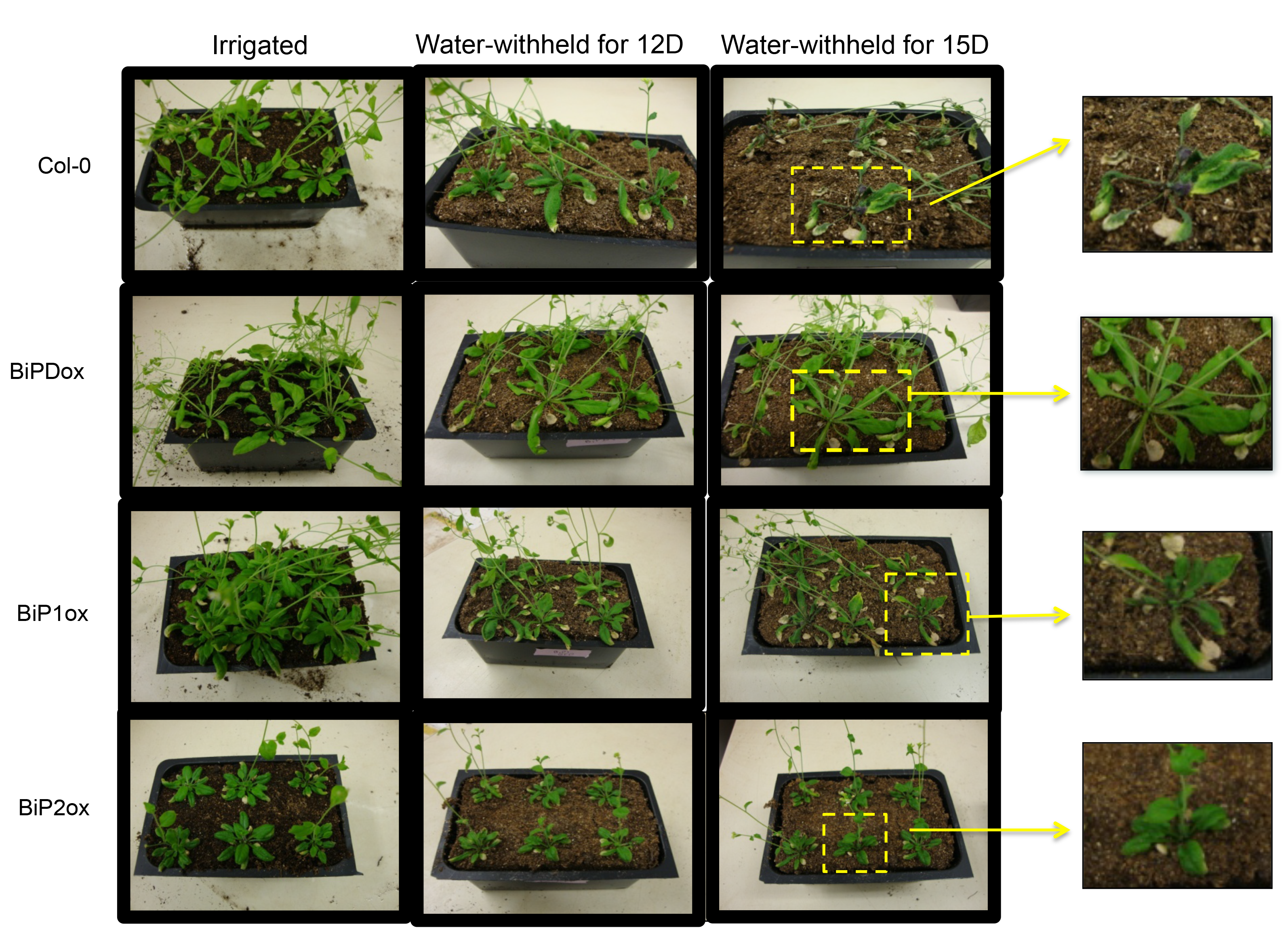

Supplement: Additional file 8: — Enhanced accumulation of Arabidopsis BiP confers tolerance to drought. Arabidopsis plants, genotypes Col–0, BiPDox, BiP1ox and BiP2ox lines were grown in soil and water stress was induced by withholding irrigation for 20 days. Photography was taken at the indicated days after withholding irrigation. (TIF 6777 kb) [file 12870_2016_843_MOESM8_ESM.tif]
