# Supplementary material for: Functional and regulatory conservation of the soybean ER stress-induced DCD/NRP-mediated cell death signaling in plants
Source: BMC Plant Biol. 2016 Jul 12;16:156. doi: 10.1186/s12870-016-0843-z (PMC4943007; doi:10.1186/s12870-016-0843-z)
Supplement: Additional file 9: — Unfolded protein response elements (UPRE) on the promoter of development cell death (DCD)/N–rich proteins (NRPs). (DOCX 58 kb) [file 12870_2016_843_MOESM9_ESM.docx]

**Additional file 8. Table S1. Unfolded protein response elements (UPRE) on the promoter of development cell death (DCD)/N-rich proteins (NRPs)**

| **Gene Name** | **PlantPAN2 Matrix ID** | **Matrix Name** | **Position*** | **Strand** | **Score Hit** | **Sequence** |
| --- | --- | --- | --- | --- | --- | --- |
| GmNRP-A | TF_motif_seq_0018 | UPRE2AT* | 654 | + | 0.8 | CCACGtcaag |
| GmNRP-B | TF_motif_seq_0060 | UPRE1AT** | 2232 | - | 0.73 | actagaCCAAT |
| AtNRP1 | TF_motif_seq_0018 | UPRE2AT | 361 | + | 0.9 | CCACGtcata |
| AtNRP1 | TF_motif_seq_0060 | UPRE1AT | 964 | + | 0.73 | ATTGGttcaac |

*XBP1 binding site-like sequence found in the plant UPRE

**ERSEII-like sequence found in the plant UPRE
