# Supplementary material for: Functional and regulatory conservation of the soybean ER stress-induced DCD/NRP-mediated cell death signaling in plants
Source: BMC Plant Biol. 2016 Jul 12;16:156. doi: 10.1186/s12870-016-0843-z (PMC4943007; doi:10.1186/s12870-016-0843-z)
Supplement: Additional file 10: — Primers used for cloning and quantitative RT–PCR. (DOCX 126 kb) [file 12870_2016_843_MOESM10_ESM.docx]

|  **Additional file 8. Table S2. Primers used for qRT-PCR analysis and for cloning** | | | |
| --- | --- | --- | --- |
| Name | Sequence 5'-3' | Target | Locus |
| qRT- Actin Fwd | GGTAACATTGTGCTCAGTGG TGG | Actin 2 | At3g187 80 |
| qRT-Actin Rvs | AACGACCTTAATCTTCATGC TGC | 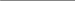 | 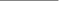  |
| qRT-UBQ5 Fwd | GACGCTTCATCTCGTCC | UBQ5 | At3g622 50 |
| qRT-UBQ5 Rvs | GTAAACGTAGGTGAGTCCA |  |   |
| qRT-AtNRP1 Fwd | CAAACGCCAGCTTTTCGGAT TG | AtNRP1 | At5g420 50  |
| qRT-AtNRP1 Rvs | TGAGCACGCTCTTCTTGCTTT CA |  |  |
| qRT-ANAC036 Fwd | TCCTCTTTCGTCTTCCGAGA | ANAC036 | At2g170 40   |
| qRT-ANAC036 Rvs: | TGCATTTGGATCTTGTTTGC |  |  |
| qRT-CNX1 Fwd | ATGAGACAACGGCAACTATT TTCC | CNX1 | At5g617 90 |
| qRT-CNX1 Rvs | CCATAATCCTCATGTCCTTC ACT |  |  |
| qRT-VPEg Fwd | CTGCTGGGCAACCTCTAGTC | VPEg | At4g32940 |
| qRT- VPEg Rvs | CGTACTGAGACAGCGATCCA | 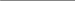 | 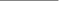  |
| NRP-B Fwd: | AAAAAGCAGGCTTCACA ATGGAGAATAATAAT | NRP-B | Glyma08g0284 0 |
| NRP-B Rvs: | AGAAAGCTGGGTCTTTA TGCCGGCAAAGCCTT |  |  |
| NRP-A Fwd | AAAAAGCAGGCTTCACA ATGGACAACAACAATG | NRP-A | Glyma20g1610 0 |
| NRP-A Rvs | AGAAAGCTGGGTCTTCTATGCTGGAATGGCTTT |  |  |
| BiPD Fwd | AAAAAGCAGGCTTCACAATGGCTGGCTCGTGG | BiPD | Glyma05g3662 0 |
| BiPD Rvs | AGAAAGCTGGGTCTTCTAGAGCTCATCGTGAGA |  |  |
| ANAC036 Fwd | AAAAAGCAGGCTTCACAATGGTTTACGGTAAGAG | ANAC036 | At2g17040 |
| ANAC036 Rvs | AGAAAGCTGGGTCTTCCA TATATGTTAATATTGG |  |  |
| AtNRP1 Fwd | AAAAAGCAGGCTTCACA ATGGAGTATAATAAC | AtNRP1 | At5g42050 |
| AtNRP1 Rvs | AGAAAGCTGGGTCTTTC AAGGGTTTTGGTCAGC |  |  |
| AtNRP2 Fwd | AAAAAGCAGGCTTCACA AGCTTCTGGCAATTA | AtNRP2 | At3g27090 |
| AtNRP2 Rvs | AGAAAGCTGGGTCTTCC AATATATGTTAACTATTGG |  |  |
| BiP1 Fwd | AAAAAGCAGGCTTCATGGCTCGCTCGTTTGG | BiP1 | At5g28540  |
| BiP1 Rvs | AGAAAGCTGGGTCCGAGCTCATCGTGAGACTCATG |  |  |
| BIP2 Fwd | AAAAAGCAGGCTTCATGGCTCGCTCGTTTGG | BiP2 | At5g42020 |
| BiP2 Rvs | AGAAAGCTGGGTCCTAGAGCTCATCGTGAGACTCATG |  |  |
| promAtNRP1 Fwd | GGGGACAACTTTGTATAGAAAAGTTGCCTCGTCA TGATTGGAAGATTG | AtNRP1 | At5g42050 |
| promAtNRP1 Rvs | GGGGACTGCTTTTTTGTACAAACTTGCCTCTAACTC TCTGATTGATCTG |  |  |
| promAtNR P2 Fwd | AAAAAGCAGGCTTCACA CTATACACTAGAGTATG ACTTTTG | AtNRP2 | At3g27090 |
| promAtNR P2 Rvs | AGAAAGCTGGGTCTTTC TAGTTTGCTCAGCTAGCT TC |  |  |
| promANAC 036 Fwd | AAAAAGCAGGCTTCACA AGATGACACTTCAACTTGAATG | ANAC036 | At2g17040 |
| promANAC 036 Rvs | AGAAAGCTGGGTCTTGTTCTTGAGGTAGAAATCAAGAAG |  |  |
| VPE-gama Fwd | AAAAAGCAGGCTTCACA ATGGCCACAACGATGACACGTG | VPE-gama | At4g32940 |
| VPE-gama Rvs | AGAAAGCTGGGTCTTTG CACTGAATCCACGGTTAAGC | 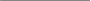 | 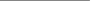 |
